# Assessing the impact of intermediate care interventions on healthcare and patient-related outcomes: a systematic overview of systematic reviews

**DOI:** 10.1186/s12913-025-13604-w

**Published:** 2025-11-03

**Authors:** Anastasios Bastounis, Burak Kundakci, Amber Muhinyi, Katherine Jones, Christopher Carroll, Andrew Booth

**Affiliations:** Sheffield Centre for Health and Related Research (SCHARR), Division of Population Health, School of Medicine and Population Health, University of Sheffield, Regent Court, 30 Regent Street, Sheffield, S1 4DA UK

**Keywords:** Emergency, Intermediate care, Overcrowding, Overview, Umbrella review

## Abstract

**Introduction:**

Intermediate care interventions are widely used to support patients transitioning between hospital and home, aiming to reduce the burden on acute healthcare services. The effectiveness however of such interventions remains unclear. Previous reviews have highlighted mixed findings, with limited high-quality evidence available to guide policy and practice.

**Aim & objectives:**

This overview aims to assess the impact of intermediate care interventions on key outcomes such as hospital length of stay (LOS), emergency department (ED) visits, readmissions, mortality, quality of life (QoL), and healthcare costs in developed countries. This overview has two key objectives: (i) to map existing widely-disseminated intermediate care interventions, and (ii) summarise evidence regarding the effectiveness of such interventions.

**Methods:**

We conducted a comprehensive search of eight databases up to February 2024. Systematic reviews with or without meta-analyses assessing intermediate care interventions in OECD developed countries were included. Two reviewers independently assessed study quality using AMSTAR 2. Data on length of stay (LOS), ED visits, readmissions, mortality, quality of life (QoL), and costs were extracted and synthesised.

**Results:**

Twenty-two reviews (570 unique primary studies) were included. Consistent evidence showed transitional care interventions (TCIs), rapid response teams, and virtual wards (VWs) were effective in reducing in-hospital LOS and ED/hospital readmissions. TCIs, person- and family-centred care, VWs, and telephone-based interventions were generally effective in lowering subsequent ED visits. Limited evidence supported effectiveness in reducing mortality, with VWs showing the most promise. No clear evidence was found for improving QoL. Cost-effectiveness findings were inconclusive. Most reviews reported low overall confidence ratings, and reviews reported that about half of primary studies were rated high risk of bias.

**Discussion:**

While intermediate care interventions show promise in addressing ED overcrowding, the generally low-certainty evidence highlights the need for further high-quality research. Future studies should focus on long-term effectiveness, cost-effectiveness, and identifying the most effective intervention components across different patient populations.

**Systematic review registration:**

PROSPERO CRD42024502585.

**Supplementary Information:**

The online version contains supplementary material available at 10.1186/s12913-025-13604-w.

## Introduction

The mismatch between demands and NHS resources can lead to delayed ambulatory responses and increased length of stay (LOS) at emergency departments (EDs) [1]. Intermediate care interventions could prove effective as part of efforts to improve patient flow, reduce ED visits, and enhance hospital discharge processes [2]. Intermediate care interventions constitute a type of system-wide healthcare service that aims to promote patients’ independence by assisting them with recovery post-discharge from acute inpatient services or by avoiding acute hospital admissions [3]. Their main aims are: (i) to assist patients, who are facing increased difficulty, in remaining at home, (ii) to assist patients with their recovery, (iii) to reduce the likelihood of unnecessary hospital visits, and (iv) to expedite patients’ discharge [4]. Intermediate care interventions can be delivered by multi-disciplinary teams at home, specialised intermediate care centres, or other community settings and may target adults who are at-risk of admission to residential care [5].

Intermediate care comprises diverse multi-component interventions, each being context-sensitive and dependent on the availability of organisational resources. Numerous systematic reviews have assessed the impact of different intermediate care interventions, whilst overviews of reviews have found inconsistent findings regarding the impact of such interventions on healthcare- and patient-related outcomes [6–8]. More specifically, pharmacy-supported interventions at transitions of care have shown inconsistent effects regarding the effectiveness of these interventions in reducing ED visits and hospitalisation rates [6], while in-hospital interventions, such as acute care geriatric units and post-fall interventions, were not found to reduce readmissions to acute care [7]. These inconsistent findings can be accounted for by the highly heterogeneous interventions assessed as well as the highly heterogeneous patient groups wherein these interventions are implemented. Assessing the impact of intermediate care on key utilisation outcomes is critical, as better performance in these domains (e.g., fewer ED visits, lower mortality, shorter LOS) can reduce healthcare costs in the long term [7]. This is particularly salient because transitions of care are a high-risk period for system and process failures (e.g., medication discrepancies), which can increase costs. It is therefore important to assess the effects of intermediate care interventions on patients’ health-related quality of life (HRQoL) alongside their economic impact over time. To date, however, there is a paucity of data on the costs of implementing such interventions and on their impact on patients’ HRQoL [6, 8].

Given the abundance of data on healthcare utilisation outcomes, it is critical to identify evidence-based intermediate care interventions to be rolled out at scale. Until now, overviews of reviews have focussed on specific types of interventions or interventions delivered exclusively in healthcare settings. We need therefore to conduct a thorough mapping of intermediate care interventions irrespective of their type and the population where these are implemented. Overviews of reviews are particularly well-suited to provide a thorough summary of the body of evidence regarding complex interventions’ effectiveness across populations and settings [9]. That said, an overview of reviews targeting intermediate care interventions holistically will provide us with a sound grasp regarding the most widely-disseminated and effective interventions across settings and patient populations.

Thus, this overview seeks to present the current body of evidence regarding the impact of intermediate care interventions on a wide range of healthcare- and patient-related outcomes. The aim of this overview is twofold: (i) to provide a mapping of the intermediate care interventions across patient groups, outcomes, and settings and (ii) to summarise the evidence regarding the effectiveness of such interventions.

## Methods

This study applied Cochrane guidance for the conduct of overviews of reviews [9] and reported following the preferred reporting items for overviews of reviews (PRIOR) statement [10]. This overview constitutes part of a larger systematic review on high-impact interventions in emergency settings [11]. A prospective protocol for this overview has been previously published in PROSPERO [CRD42024502585].

### Eligibility criteria

Eligible participants were adult patients irrespective of their gender. Paediatric populations were excluded. Any intermediate care intervention implemented at healthcare, residential or home settings was considered eligible for inclusion. Given that intermediate care services permeate the whole discharge process, interventions that are related to any type of intermediate care service (i.e., reablement, crisis response, home/bed-based)^1^ were eligible for inclusion. A set of terms relevant to intermediate care, such as acute hospital, emergency department (ED), community hospital, residential care home, nursing home, stand-alone intermediate care facility, independent sector/local authority facility, step-down, (non)bed-based settings, patient home-based, person-centred approach/risk-taking, and reablement, were all considered eligible for inclusion. Medication reconciliation, exclusively triage-based interventions, generic lean management, clinical-based or disease-specific interventions were excluded. Systematic reviews with or without (network)meta-analyses were considered eligible for inclusion. Scoping and narrative reviews were excluded; however, reviews labelled as ‘scoping’ but having followed a systematic approach (i.e., providing a transparent presentation of all stages underpinning the review process) with a clearly-demarcated research question were considered eligible for inclusion. To maximise homogeneity in the types of service provided within healthcare systems, focus was on primary studies conducted in OECD high-income countries [12]. The outcomes of interest in this overview were: (i) waiting time at EDs/hospital/inpatient, expressed as LOS, (ii) ED visits, (iii) ED/hospital readmissions, (iv) mortality, (v) quality of life (QoL) and (vi) cost. Clinical and disease-specific outcomes were excluded. Manuscripts published in peer-reviewed journals and government/public health agency reports were considered eligible for inclusion. Only studies published in English were eligible for inclusion. Posters and conference abstracts were excluded.

### Search strategy

A comprehensive search strategy was developed and run across nine databases up to February 2024 (Medline, EMBASE, APA PsycInfo, CINAHL, COCHRANE CDSR & CENTRAL, Web of Science Core Collection, Epistemonikos, HMIC) to identify eligible studies (sample of the search strategies can be found in Appendix 1). A date limit of January 2018 onwards was applied because this period marginally predates the NHS Long Term Plan (2019)^2^ and is chosen as the last major milestone for UK urgent and emergency care services prior to the current recovery plan.

### Selection process, data collection process & data items

Selected studies were imported into Rayyan online software and de-duplicated [13]. Since this overview formed part of a larger systematic review intended to inform decision-making within a short timeframe, we adopted rapid-review methods for its conduct. That said, one reviewer (ABa) screened studies for relevance based on titles/abstracts. One reviewer (ABa) screened studies for eligibility at full-text, whilst a second independent reviewer (BK) screened 50% of the selected records. Inter-rater agreement at full-text screening was assessed (Cohen’s κ). Disagreements were resolved by referring to a third arbiter (ABo/CC). A pre-piloted data extraction form was used to extract data. One reviewer (ABa) extracted data with a second independent reviewer (BK) independently extracting data from the 50% of the records. Inter-rater agreement for data extraction was not assessed. Data on the following categories was extracted: (i) first author and year, country and type of the review (plus study design of the primary studies included in the eligible reviews), (ii) date range of searches by review and study design of studies included in meta-analyses, (iii) research question by review, (iv) characteristics of the implemented interventions and targeted populations, (v) meta-analytic estimates regarding interventions’ effectiveness (e.g., odds ratios[ORs], risk ratios [RRs], mean difference [MD], standardised mean difference [SMD]) accompanied by measures of variability (e.g., 95% CIs, SDs), plus measures of heterogeneity (expressed by I^2^) and number of studies included in each meta-analysis, and (vi) narrative presentation of findings where meta-analyses were not available.

### Risk of bias assessment

The risk of bias (RoB) in the included reviews was assessed independently by two reviewers (ABa and BK/KJ/AM), using the AMSTAR 2 critical appraisal tool for systematic reviews [14]. The production of tables and graphs regarding the RoB assessment in this overview was undertaken using the R package “amstar2Vis” [15] in R version 4.3.1. The risk of bias (RoB) assessments of the primary studies included in the meta-analytic reviews within this overview were extracted by a single reviewer (ABa) and narratively synthesised to assess the quality of the evidence base and contextualise the findings.

### Synthesis methods

Outcome data was tabulated by study and intervention type and was summarised as they are reported in included reviews. Where appropriate, data was sorted and visually displayed by outcome, using forest plots. Forest plots were generated using the packages “dplyr” [16], “ggplot2” [17], and “gridExtra” [18] in R version 4.3.1. Data was narratively discussed by outcome and intervention type. Data regarding subgroup and sensitivity analyses was not extracted. The overlap of primary studies across systematic reviews included in this overview was formally assessed by estimating the corrected covered area (CCA), using the “ccaR” package in R version 4.3.1 [19].

### Reporting bias assessment & certainty assessment

Heterogeneity was evaluated at the outcome level using the I^2^ estimates. Due to the large number of primary studies included in the meta-analyses, all relevant measures were presented from each review without validating data from the corresponding primary studies. Where available, assessments of the certainty of evidence were directly extracted from the included systematic reviews by a single reviewer (ABa) and were narratively discussed.

## Results

**Fig. 1:**
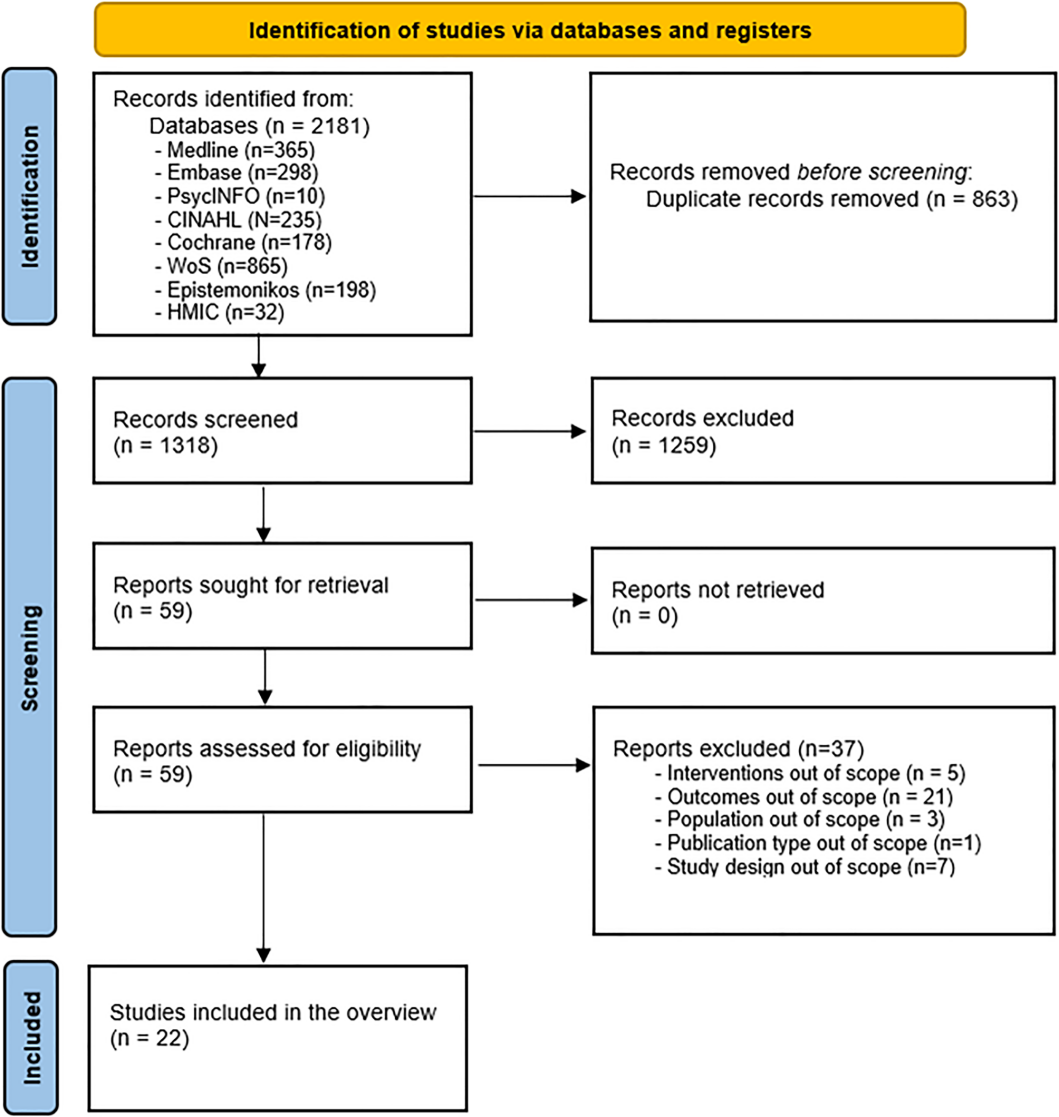
PRISMA flow diagram

A total of 2181 articles were retrieved, of which 863 were duplicates. Overall, 1,259 articles were excluded following title and abstract screening, and 37 were excluded following the full-text screen (Appendix 2); thus, 22 reviews were included in this overview (Cohen’s κ = 0.65; substantial agreement) (Fig. 1).

Most of the included reviews were systematic [20–39], while two were deemed eligible for inclusion due to their systematic approach on synthesising and reporting the data [40, 41]. Most of the included reviews (*n* = 15/22) reported a meta-analysis [20–33, 40], while the rest narratively synthesised the findings [34–39, 41].

### Characteristics of the included reviews

Included reviews were published between 2018 and 2023. The reviews were from Canada (*n* = 9), UK (*n* = 4), USA (*n* = 2), China (*n* = 3), Germany (*n* = 1), Sweden (*n* = 1), Ireland (*n* = 1), and the Netherlands (*n* = 1). All reviews used more than two databases for their searches, with Medline, EMBASE, and Cochrane Central Register of Controlled Trials and Cochrane Library being the most common searched databases. Most reviews included up to 50 primary studies, while three reviews included more than 100 studies [30, 31, 41]. Six reviews were focussed on specific patient groups [21, 26, 27, 34, 37, 40], while the rest were focussed on different types of patients at pre- to post-discharge [20, 22–25, 28–33, 35, 36, 38, 39, 41]. All reviews evaluated the impact of interventions on healthcare- and patient-related outcomes. Most reviews focussed on transitional care interventions (TCIs) with the most common being telehealth-based interventions. The most frequently assessed outcomes across reviews were ED/hospital readmissions, ED visits, LOS at hospital/ED, and mortality. Reviews characteristics table can be found in Appendix 3.

### Primary study overlap

Overall, 22 reviews reviewing 570 unique primary studies were included in the overlap assessment (Appendix 4). Overlap of included primary studies across reviews was low, with an overall corrected cover area (CCA) of 1.1%.

### Risk of bias in systematic reviews and primary studies

**Fig. 2:**
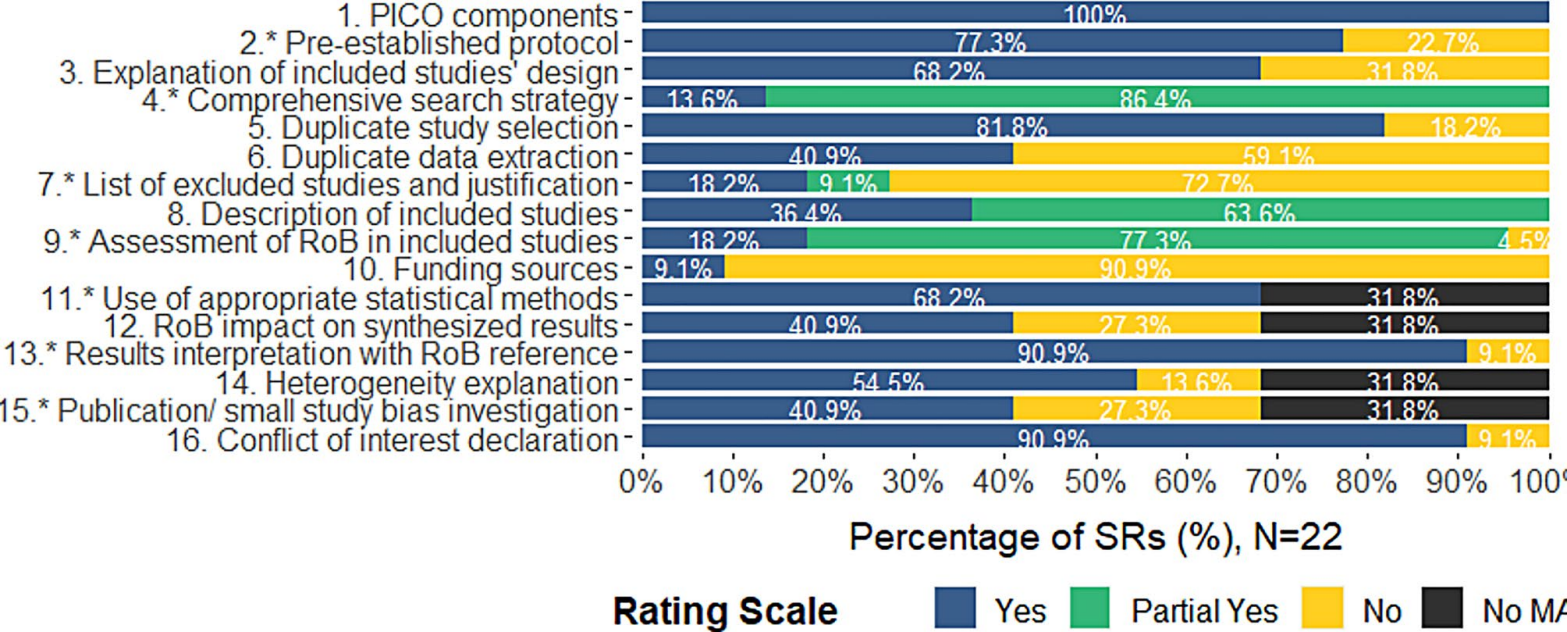
Stacked bar plot of the distribution of ratings as percentages by AMSTAR 2 domain

Overall, this sample of reviews was assessed as being of only low quality according to AMSTAR 2 criteria. Downgrades for risk of bias mostly occurred in the following AMSTAR 2 domains: (i) explanation of included studies’ design, (ii) duplicate data extraction, (iii) list of excluded studies with justification, and (iv) role of funding sources (Fig. 2). In total, 12 reviews had an overall confidence rating of low (54.5%), while seven and three reviews had an overall rating of critically low (31.8%) and moderate (13.6%), respectively. Of the critical domains, 17 out of 22 reviews had a pre-established protocol, all reviews reported a generally satisfactory literature search, while 16 out of 22 did not provide a justification for the excluded studies. Most reviews generally used satisfactory techniques to assess RoB in the included primary studies (*n* = 21), while all the reviews with a meta-analysis used appropriate statistical methods. Most reviews considered RoB assessments in the interpretation of the results (*n* = 20/22), while publication bias was not assessed in six reviews. Confidence ratings graph and table with the critical appraisal ratings by study can be found in Appendix 4.

Overall, 155/353 (43.9%) unique primary studies included in the meta-analytic reviews of this overview were rated to be of high-risk, 98 (27.75%) were rated to be of low-risk, 46 (13.1%) were rated to be of unclear risk, while 38 (10.7%), 8 (2.3%), and 8 (2.3%) of primary studies were judged to be of some, serious, and critical concerns respectively (Appendix 4). The most affected RoB domains across the primary studies were the randomisation process, allocation concealment and blinding procedures.

### Summary of results

This overview synthesised evidence from 22 reviews, encompassing 570 unique primary studies, on the effectiveness of intermediate care interventions. The main outcomes assessed were LOS, ED visits, hospital readmissions, mortality, QoL, and costs. Meta-analyses were reported for LOS (6 reviews), ED visits (9 reviews), and readmissions (12 reviews). Mortality was examined in 13 studies, with 8 reporting meta-analyses. QoL was assessed in 8 studies, 4 of which included meta-analyses. Cost-effectiveness was evaluated in 6 studies.

#### Length of stay (LOS)

**Fig. 3:**
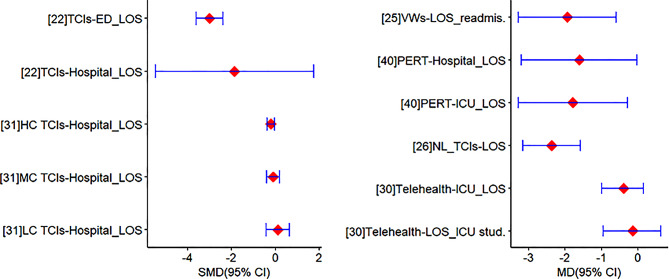
Meta-analytic estimates regarding the effectiveness of intermediate care interventions in reducing LOS*. Note. HC: high-complexity; ICU: intensive care unit; LC: low-complexity; MC: medium-complexity; MD: mean difference; NL: nurse-led; PERTs: pulmonary embolism response teams; SMD: standardised mean difference; TCIs: transitional care interventions. *Values < 0 indicate shorter LOS at ED/hospital favouring the intermediate care interventions

Overall, data regarding the effects of intermediate care interventions on LOS was drawn from 12 reviews [22, 25–27, 30, 31, 36–41] with six of them reporting meta-analyses [22, 25, 26, 30, 31, 40] (Fig. 3). Meta-analyses included from four [25] to 12 reviews [30, 31]. Meta-analytic estimates showed that high-complexity TCIs (indicated by the number of components and discharge stages) (SMD = -3 [95%CI: -3.61, -2.39], *p* < 0.05; SMD = -0.2 [95%CI: -0.38, -0.03], *p* < 0.5) [22, 31], VWs (MD = -1.94 [95%CI: -3.28, -0.6], *p* < 0.05) [25], PERTs (MD = -1.61 [95%CI: -3.21, -0.02], *p* < 0.05; MD = -1.79 [95%CI: -3.29, -0.28], *p* < 0.05) [40], and nurse-led TCIs (MD = -2.37 [95%CI: -3.16, -1.58], *p* < 0.05) [26] were effective in reducing EDs/hospital/ICU LOS, and LOS readmission following discharge. The remaining studies found that nurse-led TCIs were associated with reduced LOS [27, 36, 37], while inconsistent effects were observed regarding the impact of ED-based interventions, rehabilitation, and palliative care support [38, 39, 41].

#### ED visits

**Fig. 4:**
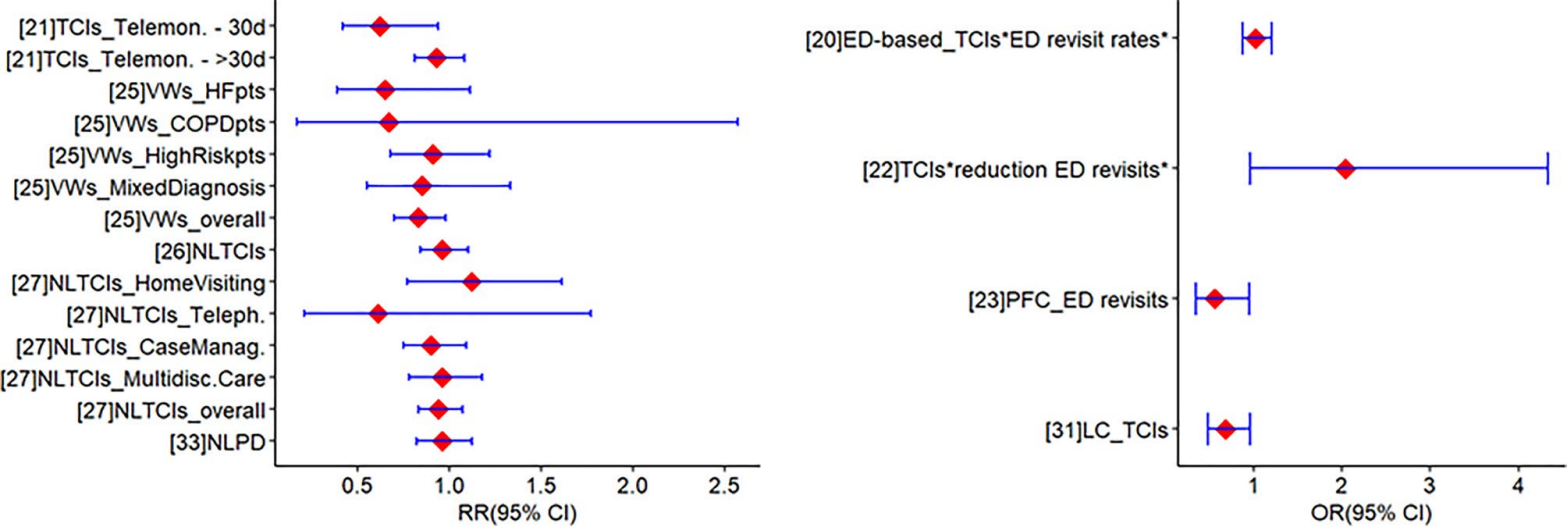
Meta-analytic estimates regarding the effectiveness of intermediate care interventions in reducing the number of (re)-visits at ED*. Note. CI: confidence interval; COPD: chronic obstructive pulmonary disease; HF: heart failure; LC: low-complexity; NL: nurse-led; NLPD: nurse-led peri-discharge; OR: odds ratio; PFC: Person- and family-centred care; RR: risk ratio; TCIs: transitional care interventions; VW: virtual ward. *Values < 1 indicate lower probability for subsequent ED visits, favouring the intermediate care interventions

Overall, data regarding the effects of intermediate care interventions on ED visits was drawn from 13 studies [20–27, 31–33, 35, 39] with nine of them reporting meta-analyses [20–23, 25–27, 31, 33] (Fig. 4). Meta-analyses included from two [21] to 41 studies [31]. Meta-analysis estimates showed that telemonitoring (RR = 0.62 [95%CI: 0.42, 0.94], *p* < 0.05) [21], PFC (OR = 0.56 [95%CI: 0.34, 0.95], *p* < 0.05) [23], VWs (RR = 0.83 [95%CI: 0.7, 0.98], *p* < 0.05) [25], and low-complexity TCIs (OR = 0.68 [95%CI: 0.48, 0.96], *p* < 0.05) [31] were effective in reducing ED visits within 30 days post-discharge. Also, ED-based TCIs were found to be effective in reducing outpatient follow-up rates (OR = 1.79 [95%CI: 1.43, 2.24], *p* < 0.05) [20]. Two reviews found that ED-based interventions were associated with reductions in ED visits [24, 35], while inconsistent findings - in terms of the direction and precision of the effects - regarding the effectiveness of TCIs and ED-based interventions were observed in two reviews [32, 39].

#### Readmissions

Overall, data regarding the effects of intermediate care interventions on ED/hospital readmissions was drawn from 20 studies [20–33, 35–39, 41] with 12 of them reporting meta-analyses [20–23, 25–29, 31–33] (Appendix 5). Meta-analyses included from four [23] to 73 studies [31]. Apart from three reviews [20, 29, 33], meta-analysis estimates showed that intermediate care interventions, namely telephone-based (RR = 0.72 [95%CI: 0.63, 0.81], *p* < 0.05) [21] and multi-component TCIs (RR = 0.63 [95%CI: 0.45, 0.88], *p* < 0.05; OR = 0.75 [95%CI: 0.62, 0.91], *p* < 0.05) [21, 32], PFC (OR = 0.56 [95%CI: 0.34, 0.95], *p* < 0.05) [23], VWs (RR = 0.91 [95%CI: 0.85, 0.98], *p* < 0.05) [25], nurse-led TCIs (RR = 0.91 [95%CI: 0.82, 0.99], *p* < 0.05; RR = 0.89, [95%CI: 0.82, 0.97], *p* < 0.05; RR = 0.78 [95%CI: 0.68, 0.89], *p* < 0.05) [26, 27], TCIs of low- and middle-complexity (OR = 0.78 [95%CI: 0.66, 0.92], *p* < 0.05; OR = 0.82 [95%CI: 0.68, 0.97], *p* < 0.05) [31], critical care outreach (RR = 0.64 [95%CI: 0.42, 0.99], *p* < 0.05; RR = 0.62 [95%CI: 0.43, 0.9], *p* < 0.05) [28] and rapid response teams (RR = 0.76 [95%CI: 0.67, 0.87], *p* < 0.05) [28], were effective in reducing the hospital readmissions within 30 days post-discharge [21–23, 25–29, 31, 32], with these effects extending to >30 days post-discharge [21, 31, 32]. Also, the implementation of TCIs was found to be associated with a reduction in hospital/ED readmissions (OR = 1.66 [95%CI: 1.18, 2.35], *p* < 0.05) [22]. The remaining reviews found that ED-based, primary-care and home-based interventions, outpatient telehealth consultations, ED-based TCIs and advanced nursing care interventions, and multi-disciplinary coaching interventions were associated with reductions in hospital readmissions [24, 30, 35, 36, 39]. Inconsistent findings regarding the effectiveness of TCIs and palliative care interventions were observed in two reviews [37, 38].

#### Mortality, quality of life (QoL) and costs

Overall, data regarding the effects of intermediate care interventions on mortality was drawn from 13 studies [21, 22, 24, 25, 28–30, 33, 36–41] with eight studies reporting meta-analyses [21, 22, 25, 28–30, 33, 40] (Appendix 5). Meta-analysis estimates showed that telemonitoring (RR = 0.62 [95%CI: 0.42, 0.94], *p* < 0.05) [21] and VWs (RR = 0.9 [95%CI: 0.82, 0.97], *p* < 0.05) [25] were associated with lower mortality rates. No evidence regarding the effects of home-based, advanced nursing and palliative care and TCIs in reducing mortality were detected [24, 36–38, 41]. Overall, data regarding the effects of intermediate care interventions on patients’ QoL was drawn from eight studies [21, 22, 25, 31, 34, 37, 38, 41] with four of them reporting meta-analyses [21, 22, 25, 31]. Meta-analysis estimates showed no evidence regarding the effectiveness of TCIs and VWs in increasing QoL across patients [21, 22, 25, 31]. The remaining reviews found no evidence regarding the effects of PFC, TCIs, palliative care interventions [34, 37, 38], while education coaching interventions were found to be generally effective [41]. Overall, six studies assessed the cost related to the implementation of intermediate care interventions [20, 24, 30, 36, 37, 41]. Inconsistent findings - in terms of the magnitude and precision - regarding the cost-effectiveness of intermediate care interventions were found, while primary care interventions (MD=-$4119 [95%CI: -$7935, -$302], *p* < 0.05) [24] and TCIs with assisted technology tools seemed to be cost-effective [36, 37, 41].

### Assessment of reporting bias and certainty of evidence

All reviews were generally well-reported on their pre-defined analysis and used appropriate methods of analysing the data. Most of the primary studies included in the reviews were deemed to be of high risk of bias at least in one domain. In four out of six reviews reporting meta-analysis of LOS data, the level of heterogeneity was higher than 75%. In three out of nine reviews reporting meta-analysis of ED-visits data, the level of heterogeneity was over 70%. In three out of 12 reviews reporting meta-analysis of readmissions data, the level of heterogeneity was over 70%. In three out of eight reviews reporting meta-analysis of mortality data, the level of heterogeneity was over 75%.

No Grading of Recommendations Assessment, Development and Evaluation (GRADE) assessment was provided for LOS data. Three out of nine reviews that meta-analysed ED-visits data reported GRADE certainty of evidence assessments. In two reviews the certainty of evidence was judged to be low [20, 21], while in one review the certainty of evidence was judged to be high [33]. Four out of 12 reviews reporting meta-analyses of readmissions data provided GRADE certainty of evidence assessments. The certainty of evidence was judged to be low [20], moderate [21, 33], and very low [28]. Four out of eight studies reporting meta-analysis on mortality data provided certainty of evidence assessments with those assessments ranging from very low [28] to moderate [21, 30, 33].

## Discussion

This overview included 22 reviews representing 570 unique primary studies and showed consistent evidence of the effectiveness of TCIs, rapid response teams, and VWs in reducing in-hospital LOS and ED/hospital readmissions. Relatively consistent evidence was found regarding the effectiveness of TCIs, PFC, VWs, and telephone-based interventions in lowering the probability of subsequent ED-visits after discharge. Little evidence was found regarding the effectiveness of intermediate care interventions in lowering the probability of mortality, with any positive effects being more prominent in VWs (including telemonitoring). No evidence was found regarding the effectiveness of intermediate care interventions in increasing patients’ QoL, while mixed findings were found on the cost-effectiveness of such interventions. A paucity of data was evident on the long-term effectiveness (> 30 days post-discharge) of intermediate care interventions, while there was an overrepresentation of TCIs across reviews.

The implementation of intermediate care interventions could streamline the discharge process and alleviate the overcrowding in EDs/hospital settings by reducing ED and hospital LOS and decreasing the probability of readmissions. However, we generally found low-certainty evidence for the impact of most interventions, while most of the included reviews had a low overall confidence rating and approximately half of the included primary studies were rated high in RoB. As such, further research is needed to better evaluate the impact of such interventions. Cross-comparison of the most promising intermediate care interventions within clustered controlled designs could help to evaluate their effectiveness in specific patient populations. This becomes more apparent in the case of TCIs, given that this intervention type was found to be the most widely-implemented across patient groups. Further research on the most effective components of such interventions (e.g., TCIs and VWs), will facilitate the development and implementation of more tailored approaches, considering the organisational resources and patient needs. Future research could also assess long-term effects of such interventions to permit better estimates of their cost-effectiveness and sustainability. This overview included data from studies conducted exclusively in high-income countries to reduce, as far as possible, variability in service provision and organisational context; the findings are therefore not directly applicable to healthcare settings in low- and middle-income countries (LMICs). However, future research on intermediate care in LMICs can capitalise on these findings, in that several intervention families synthesised here could be adapted with appropriate local contextualisation. For instance, lower-complexity transitional-care components (e.g., structured discharge planning, patient/caregiver education) are likely to be more feasible than resource-intensive models in LMICs.

### Strengths and limitations

This overview has several strengths. First, this overview employed state of the art methods in conducting and reporting the findings. Second, to the best of our knowledge, this is the largest overview of intermediate care interventions, considering the stringent date range of searches. However, this overview inevitably has limitations. First, dual-independent screening was not conducted. However, given the number of reviews identified and comparisons with relevant overviews, it is highly unlikely that any significant studies - those that could have altered the direction of the findings- were missed. Second, the data extracted from the reviews and the included primary studies was not cross-checked for accuracy or missing information. Third, the level of heterogeneity was generally high in most of the comparisons, while subgroup analysis was not undertaken. Fourth, certainty of evidence was directly extracted from the included reviews. Fifth, the findings of this overview are drawn from reviews that have primarily focused on OECD countries, which limits the generalisability of our results to developing and low-income countries. Sixth, the searches were conducted in February 2024 and were not subsequently updated, so some potentially eligible reviews may have been missed. However, given the number of included reviews and the abundance of data on healthcare utilisation outcomes, we are confident that any missed reviews are unlikely to substantively change the direction or magnitude of our findings.

## Conclusion

This overview of systematic reviews provides evidence that intermediate care interventions can be effective in addressing key challenges in emergency and acute care settings. Transitional care interventions (TCIs), rapid response teams, and virtual wards (VWs) consistently demonstrated effectiveness in reducing hospital length of stay (LOS) and readmission rates. TCIs, person- and family-centred care, VWs, and telephone-based interventions also showed promise in reducing subsequent ED visits. However, evidence for impact on mortality and quality of life was limited, and cost-effectiveness findings were inconclusive. While these interventions show potential for alleviating ED overcrowding and improving patient flow, the generally low-certainty evidence underscores the need for further high-quality research to optimise implementation and evaluate their long-term effectiveness across diverse patient populations and healthcare settings.

## Supplementary Information

Below is the link to the electronic supplementary material.

Supplementary Material 1

## Data Availability

All data generated or analysed during this study are included in this published article [and its supplementary information files].

## Footnotes

1 NICE. Understanding intermediate care, including reablement. https://www.nice.org.uk/about/nice-communities/social-care/quick-guides/understanding-intermediate-care.

2 NHS. The NHS long term plan. 2019. https://www.longtermplan.nhs.uk/.
