# Supplementary material for: Assessing the impact of intermediate care interventions on healthcare and patient-related outcomes: a systematic overview of systematic reviews
Source: BMC Health Serv Res. 2025 Nov 3;25:1435. doi: 10.1186/s12913-025-13604-w (PMC12581353; doi:10.1186/s12913-025-13604-w)
Supplement: Supplementary file 1 — Supplementary Material 1 [file 12913_2025_13604_MOESM1_ESM.docx]

**Appendices**

**Title:** Assessing the impact of intermediate care interventions on healthcare and patient-related outcomes: An umbrella review. PROSPERO 2024 CRD42024502585, Available from: <https://www.crd.york.ac.uk/prospero/display_record.php?ID=CRD42024502585>

**Authors:** Bastounis, A., Kundakci, B., Muhinyi, A., Jones, K., Carroll, C., & Booth, A.

**Appendix Table 1.**  Search syntax for Tier I searches

| **Ovid MEDLINE(R) Epub Ahead of Print and In-Process, In-Data-Review & Other Non-Indexed Citations <February 23, 2024>** | |
| --- | --- |
| #1 | "urgent community response".ab,ti. (1) |
| #2 | ucr.ab,ti. (850) |
| #3 | "rapid response service* ".ab,ti. (31) |
| #4 | hrrs.ab,ti. (289) |
| #5 | (community adj3 urgent care).ab,ti. (35) |
| #6 | "urgent home care".ab,ti. (1) |
| #7 | After-Hours Care/ (2116) |
| #8 | ("after hours" adj3 (service* or care or clinic* or centre* or center*)).ab,ti. (504) |
| #9 | ("out of hours" adj3 (service* or care or clinic* or center* or centre*)).ab,ti. (1148) |
| #10 | ((gp* or "general practice* or general practitioner*") adj3 ("out of hours" or co?operative* or collaborative*)).ab,ti. (479) |
| #11 | "on site paramedic* ".ab,ti. (3) |
| #12 | Access to Primary Care/ (35) |
| #13 | "access to primary care".ab,ti. (1539) |
| #14 | ("rural health" adj (clinic* or centre* or center*)).ab,ti. (1023) |
| #15 | 1 or 2 or 3 or 4 or 5 or 6 or 7 or 8 or 9 or 10 or 11 or 12 or 13 or 14 (7046) |
| #16 | ((ED or emergency or A&E) adj5 (wait* or time* or duration or length or minute* or hour*)).ab,ti. (20750) |
| #17 | (ambulance* and ("response time*" or delay* or "waiting time*")).ab,ti. (1625) |
| #18 | 16 or 17 (22076) |
| #19 | 15 and 18 (434) |
| #20 | limit 19 to yr="2018 -Current" (139) |

**Appendix Table 2.** Search syntax for Tier II-III searches

| **Ovid MEDLINE(R) ALL <1946 to February 14, 2024>** | |
| --- | --- |
| #1 | *Emergency Service, Hospital/ 47539 |
| #2 | *Emergency Medical Services/ 36394 |
| #3 | *Emergency Medicine/ 11957 |
| #4 | (emergency adj2 service*).ab,ti. 22064 |
| #5 | "emergency care".ab,ti. 11778 |
| #6 | "urgent care".ab,ti. 3368 |
| #7 | "emergency department* ".ab,ti. 130878 |
| #8 | "accident and emergency".ab,ti. 5047 |
| #9 | casualty.ab,ti. 6808 |
| #10 | exp *Critical Care/ 38577 |
| #11 | *intensive care units/ or *recovery room/ 29500 |
| #12 | or/1-11 272791 |
| #13 | Intermediate Care Facilities/ or (intermediate care or IMCU or IMCUs).mp. 2379 |
| #14 | (step-up or step-down or stepup or stepdown).mp. 7622 |
| #15 | *subacute care/ 913 |
| #16 | ((post-acute or sub-acute or subacute) adj3 care).ti. 852 |
| #17 | *transitional care/ 1132 |
| #18 | 13 or 14 or 15 or 16 or 17 12442 |
| #19 | 12 and 18 790 |
| #20 | *"continuity of patient care"/ or *patient transfer/ 16759 |
| #21 | (outreach or out-reach or ((followup or follow* up) adj3 care)).mp. 31380 |
| #22 | *home care services, hospital-based/ or *rehabilitation centers/ 5877 |
| #23 | 21 or 22 37194 |
| #24 | 20 and 23 673 |
| #25 | 12 and 24 113 |
| #26 | 19 or 25 899 |
| #27 | limit 26 to yr="2018 - 2024" 365 |

**Appendix Table 3.** Search syntax for Tier II-III

| **Embase <1974 to 2024 Week 06>** | |
| --- | --- |
| #1 | exp *emergency medicine/ 30847 |
| #2 | exp *emergency health service/ 101369 |
| #3 | exp *emergency care/ 19156 |
| #4 | (emergency adj2 service*).ab,ti. 30586 |
| #5 | "emergency care".ab,ti. 15612 |
| #6 | "urgent care".ab,ti. 5599 |
| #7 | "emergency department* ".ab,ti. 201197 |
| #8 | "accident and emergency".ab,ti. 6652 |
| #9 | casualty.ab,ti. 8105 |
| #10 | or/1-9 316746 |
| #11 | intermediate care unit/ or (intermediate care or IMCU or IMCUs).mp. 3261 |
| #12 | (step-up or step-down or stepup or stepdown).mp. 12308 |
| #13 | *subacute care/ 648 |
| #14 | ((post-acute or sub-acute or subacute) adj3 care).ti. 1151 |
| #15 | *transitional care/ 2633 |
| #16 | 11 or 12 or 13 or 14 or 15 19484 |
| #17 | 10 and 16 1103 |
| #19 | limit 17 to yr="2018 -Current" 567 |
| #20 | limit 18 to embase 298 |

**Appendix Table 4.** Search syntax for Tier II-III

| **APA PsycInfo <1806 to February Week 2 2024>** | |
| --- | --- |
| #1 | exp *emergency medicine/ 469 |
| #2 | (emergency adj2 service*).ab,ti. 3473 |
| #3 | "emergency care".ab,ti. 1082 |
| #4 | "urgent care".ab,ti. 414 |
| #5 | "emergency department* ".ab,ti. 11531 |
| #6 | "accident and emergency".ab,ti. 517 |
| #7 | casualty.ab,ti. 844 |
| #8 | or/1-7 16520 |
| #9 | intermediate care unit/ or (intermediate care or IMCU or IMCUs).mp. 494 |
| #10 | (step-up or step-down or stepup or stepdown).mp. 1401 |
| #11 | ((post-acute or sub-acute or subacute) adj3 care).ti. 111 |
| #12 | 9 or 10 or 11 2002 |
| #13 | 8 and 12 20 |
| #14 | limit 13 to yr="2018 -Current" 10 |

**Appendix Table 5.** Search syntax for Tier II-III

| **CINAHL** | |
| --- | --- |
| #1 | S5 AND S9 235 |
| #2 | S5 AND S9 544 |
| #3 | S6 OR S7 OR S8 7,404 |
| #4 | (step-up or step-down or stepup or stepdown ) OR ( (post-acute or sub-acute or subacute) n3 care) ) 6,607 |
| #5 | IMCU or IMCUs 34 |
| #6 | "intermediate care" OR (MH "Subacute Care") 2,889 |
| #7 | S1 OR S2 OR S3 OR S4 198,170 |
| #8 | ( "emergency care" or "urgent care" or casualty ) OR ( "accident and emergency" or "emergency department" ) 115,893 |
| #9 | (MH "Intensive Care Units") OR (MH "Critical Care") 64,983 |
| #10 | (MH "Emergency Care+") 38,764 |
| #11 | (MH "Emergency Medicine") 13,174 |

**Appendix Table 6.** Search syntax for Tier II-III

| **COCHRANE (CDSR & CENTRAL)** (Date Run: 16/02/2024) | |
| --- | --- |
| #1 | MeSH descriptor: [Emergency Service, Hospital] explode all trees 3900 |
| #2 | MeSH descriptor: [Emergency Medical Services] explode all trees 6050 |
| #3 | MeSH descriptor: [Emergency Medicine] explode all trees 377 |
| #4 | (emergency near/2 service*):ti,ab,kw 7278 |
| #5 | ("emergency care"):ti,ab,kw 1981 |
| #6 | ("urgent care"):ti,ab,kw 490 |
| #7 | ("emergency department"):ti,ab,kw 12579 |
| #8 | ("accident and emergency"):ti,ab,kw 322 |
| #9 | (casualty):ti,ab,kw 250 |
| #10 | MeSH descriptor: [Critical Care] explode all trees 3065 |
| #11 | MeSH descriptor: [Intensive Care Units] explode all trees 5967 |
| #12 | #1 or #2 or #3 or #4 or #5 or #6 or #7 or #8 or #9 or #10 or #11 27045 |
| #13 | MeSH descriptor: [Intermediate Care Facilities] explode all trees 17 |
| #14 | ("intermediate care" or IMCU or IMCUs):ti,ab,kw 244 |
| #15 | ("step up" or stepup or "step down" or stepdown):ti,ab,kw 1941 |
| #16 | MeSH descriptor: [Subacute Care] explode all trees 33 |
| #17 | MeSH descriptor: [Transitional Care] explode all trees 144 |
| #18 | ((postacute or post-acute or subacute or sub-acute) near/3 care):ti,ab,kw 412 |
| #19 | #13 or #14 or #15 or #16 or #17 or #18 2715 |
| #20 | #12 and #19 178 (8 reviews, 170 trials) |

**Appendix Table 7.** Search syntax for Tier II-III

| **Web of Science Core Collection** | |
| --- | --- |
| #1 | emergency near/2 (service* OR medic* OR care OR room OR department OR ward OR accident) (Topic) OR "urgent care" (Topic) OR casualty OR "critical care" OR "intensive care" (Topic) - Results: 484335 |
| #2 | ((TS=("intermediate care" OR IMCU OR IMCUs)) OR TS=((subacute OR sub-acute OR postacute OR post-acute) near/2 care)) OR TS=(stepup OR "step up" OR stepdown OR "step down") - Results: 23943 |
| #3 | #2 AND #1 - Results: 1687 |
| #4 | #3 AND (2018 OR 2019 OR 2020 OR 2021 OR 2022 OR 2023 OR 2024) - Results: 865 |

**Appendix Table 8.** Search syntax for Tier II-III

| **Epistemonikos** |
| --- |
| (title:((title:("emergency service" OR "emergency services" OR "emergency medicine" OR "emergency medical" OR "emergency care" OR "emergency room" OR "emergency department" OR "emergency ward" OR "accident AND emergency" OR "urgent care" OR casualty OR "critical care" OR "intensive care") OR abstract:("emergency service" OR "emergency services" OR "emergency medicine" OR "emergency medical" OR "emergency care" OR "emergency room" OR "emergency department" OR "emergency ward" OR "accident AND emergency" OR "urgent care" OR casualty OR "critical care" OR "intensive care"))) OR abstract:((title:("emergency service" OR "emergency services" OR "emergency medicine" OR "emergency medical" OR "emergency care" OR "emergency room" OR "emergency department" OR "emergency ward" OR "accident AND emergency" OR "urgent care" OR casualty OR "critical care" OR "intensive care") OR abstract:("emergency service" OR "emergency services" OR "emergency medicine" OR "emergency medical" OR "emergency care" OR "emergency room" OR "emergency department" OR "emergency ward" OR "accident AND emergency" OR "urgent care" OR casualty OR "critical care" OR "intensive care")))) AND (title:((title:("intermediate care" OR IMCU OR IMCUs OR "subacute care" OR "sub-acute care" OR "postacute care" OR "post-acute care" OR stepup OR "step up" OR stepdown OR "step down") OR abstract:("intermediate care" OR IMCU OR IMCUs OR "subacute care" OR "sub-acute care" OR "postacute care" OR "post-acute care" OR stepup OR "step up" OR stepdown OR "step down"))) OR abstract:((title:("intermediate care" OR IMCU OR IMCUs OR "subacute care" OR "sub-acute care" OR "postacute care" OR "post-acute care" OR stepup OR "step up" OR stepdown OR "step down") OR abstract:("intermediate care" OR IMCU OR IMCUs OR "subacute care" OR "sub-acute care" OR "postacute care" OR "post-acute care" OR stepup OR "step up" OR stepdown OR "step down"))))  * Limited to 2018-2024 = 198 results |

**Appendix Table 9.** List of excluded studies with reasons

| **Citation** | **Title** | **Reason for exclusion** |
| --- | --- | --- |
| Barbosa_2023 | Comparative Effectiveness of Coronary CT Angiography and Standard of Care for Evaluating Acute Chest Pain: A Living Systematic Review and Meta-Analysis | Intervention out of scope |
| Burch_2023 | The Cost Effectiveness of Coronary CT Angiography and the Effective Utilization of CT-Fractional Flow Reserve in the Diagnosis of Coronary Artery Disease | Intervention out of scope |
| Falsetti_2021 | Clinical Method Applied to Focused Ultrasound: The Case of Wells' Score and Echocardiography in the Emergency Department: A Systematic Review and a Meta-Analysis | Outcomes out of scope |
| Heuer_2022 | Simulation-Based Training Within Selected Allied Health Professions: An Evidence-Based Systematic Review | Intervention out of scope |
| Chou_2020 | Opioid Treatments for Chronic Pain | Intervention out of scope |
| Anderson dos, Santos_2021 | Telehealth as a health education tool in the fight against the new Coronavirus: a systematic review | Outcomes out of scope |
| deSouza_2022 | Rapid tranquilization of the agitated patient in the emergency department: A systematic review and network meta-analysis | Outcomes out of scope |
| Baumgartner_2020 | Interventions to deprescribe potentially inappropriate medications in the elderly: Lost in translation? | Outcomes out of scope |
| Brady_2018 | The HEART score: A guide to its application in the emergency department | Outcomes out of scope |
| Patel_2018 | Can early warning scores identify deteriorating patients in pre-hospital settings? A systematic review | Outcomes out of scope |
| Waydhas_2018 | Intermediate care units: Recommendations on facilities and structure | Outcomes out of scope |
| Numeroso_2019 | Emergency department management of patients with syncope according to the 2018 ESC guidelines: Main innovations and aspect deserving a further improvement | Outcomes out of scope |
| Grant_2020 | Reducing preventable patient transfers from long-term care facilities to emergency departments: a scoping review | Study design out of scope |
| Cetin-Sahin_2023 | Taxonomy of Interventions to Reduce Acute Care Transfers From Long-term Care Homes: A Systematic Scoping Review | Outcomes out of scope |
| Ba_2020 | Transitional care interventions for patients with heart failure: An integrative review | Study design out of scope |
| Shilati_2023 | Transitional care programs for trauma patients: A scoping review | Outcomes out of scope |
| Stewart_2019 | An Unmet Need Meets an Untapped Resource: Pharmacist-Led Pathways for Hypertension Management for Emergency Department Patients | Publication type out of scope |
| Molitor_2022 | Occupational Therapy and the IMPACT Act: Part 1. A Systematic Review of Evidence for Fall Prevention and Reduction, Community Discharge and Reintegration, and Readmission Prevention Interventions | Intervention out of scope |
| Coffey_2019 | Interventions to Promote Early Discharge and Avoid Inappropriate Hospital (Re) Admission: A Systematic Review | Population out of scope |
| Micalleff_2022 | Defining Delayed Discharges of Inpatients and Their Impact in Acute Hospital Care: A Scoping Review | Study design out of scope |
| Hecimovic_2020 | Characteristics and outcomes of patients receiving Hospital at Home Services in the South West of Sydney | Outcomes out of scope |
| Misset_2023 | Construction of reference criteria to admit patients to intermediate care units in France: a Delphi survey of intensivists, anaesthesiologists and emergency medicine practitioners (first part of the UNISURC project) | Outcomes out of scope |
| Huntley_2020 | Determining when a hospital admission of an older person can be avoided in a subacute setting: a systematic review and concept analysis | Outcomes out of scope |
| Cadel_2021 | Initiatives for improving delayed discharge from a hospital setting: a scoping review | Study design out of scope |
| Braz_2020 | Strategies for case management in transitional care in emergency services: scoping review | Study design out of scope |
| Bayuo_2023 | Transitional and aftercare needs of persons recovering from COVID-19 using the Omaha System: A Scoping Review | Study design out of scope |
| Witting_2022 | Early Prediction of Intensive Care Admission in Emergency Department Patients With Asthma | Outcomes out of scope |
| Shah_2018 | Improving the ED-to-Home Transition: The Community Paramedic-Delivered Care Transitions Intervention-Preliminary Findings | Outcomes out of scope |
| Hager_2022 | Models of Intermediate Care Organization and Staffing at an Academic Medical Center: Considerations of an Inpatient Planning Committee | Outcomes out of scope |
| He_2019 | A systematic review of research design and modeling techniques in inpatient bed management | Outcomes out of scope |
| Rider_2018 | Transition of care from the emergency department to the outpatient setting: A mixed-methods analysis | Outcomes out of scope |
| Chen_2019 | Routine admission to step-down unit as an alternative to intensive care unit after pediatric supraglottoplasty | Population out of scope |
| Hall_2018 | Caseworker-assigned discharge plans to prevent hospital readmission for acute exacerbations in children with chronic respiratory illness | Population out of scope |
| Krause_2023 | Developing a Trauma Intermediate Care Unit | Study design out of scope |
| Melmer_2023 | Optimizing Transitions of Care and Enhancing Surgical Education on Acute Care Surgery: A Multi-Institutional Survey Study | Outcomes out of scope |
| Gillespie_2019 | Standards for the Use of Telemedicine for Evaluation and Management of Resident Change of Condition in the Nursing Home | Outcomes out of scope |
| Ticinesi_2020 | The Geriatric Patient: The Ideal One for Chest Ultrasonography? A Review From the Chest Ultrasound in the Elderly Study Group (GRETA) of the Italian Society of Gerontology and Geriatrics (SIGG) | Outcomes out of scope |

**Appendix Table 10.** Characteristics of the included studies

| **First author, year (ref.)** | **Country; total number of studies; number of (relevant) primary studies; n of databases searched; date range of searches** | **Type of reviews (study design of primary studies included in MA)** | **Aim** | **Intervention & Population** | **Findings & other outcomes*,**** |
| --- | --- | --- | --- | --- | --- |
| Aghajafari, 2020 | Canada; N=42; Relevant: 41 (Australia: 5, Belgium: 1, Canada: 8, Denmark: 1, France: 2, Sweden: 1, UK: 3, USA: 20); 4 databases; Inception – 10/2018 | SR & MA (RCTs) | To assess the effects of ED-based TCIs on ED re-visits and follow-up visits with primary care physicians. | Interventions: ED-based TCIs, such as educational support (face-to-face, video-based, or telephonic), reminders (mailed, text or telephonic), appointment scheduling, ED-based discharge instructions and case management programmes  Population: Adult patients attending ED | Meta-analysis results (OR, 95%CI)  - ED-based TCIs (studies=20; outcome: outpatient follow-up rates): **1.79 (1.43, 2.24)**, I^2^=71% (SoE: Low) (favouring UC)  - ED-based TCIs (studies=20; outcome: ED revisits rates): 1.02 (0.87, 1.20), I^2^=41% (SoE: Low)  - ED-based TCIs (studies=13; outcome: Hospital admissions after ED discharge): 0.99 (0.86, 1.14), I^2^=0% (SoE: Low)  - Cost effectiveness (studies=5): 3/5 studies found no significant differences between TCIs and CG/UC, while the remaining two studies found significant effects favouring, in the first case, the TCI group and, in the other case, the CG/UC. |
| Al Sattouf, 2022 | UK; N=13; Relevant: 8 (Australia: 2; Belgium: 1; Canada: 1; Spain: 1; USA: 3); 5 databases; 04/2012 – 04/2022 | SR & MA (RCTs) | To assess the effects of TCIs on hospital readmissions, ED visits, mortality rates, QoL. | Interventions: TCIs (home-visit programmes, structured telephone support, telemonitoring, clinic-based, educational)  Population: Adults with HF that needed hospitalisation. | Meta-analysis results (RR, 95%CI)  - Educational interventions (studies=2; outcome: 30-day readmission): 1.06(0.91, 1.24)  - Educational interventions (studies=3; outcome: >30-day readmission): 0.74(0.5, 1.09)  - Clinic-based interventions (studies=1; outcome: >30-day readmission): 0.92(0.74, 1.14)  - Telemonitoring (studies=3; outcome: 30-day readmission): 1.02(0.85, 1.23)  - Telemonitoring (studies=1; outcome: >30-day readmission): 1.03(0.93, 1.15)  - Telephone-based interventions (studies=3; outcome: >30-day readmission): **0.72(0.63, 0.81)**  - Multicomponent intervention (studies=1; outcome: 30-day readmission): **0.63(0.45, 0.88)**  - Multicomponent intervention (studies=1; outcome: >30-day readmission): **0.62(0.48, 0.81)**  Total: **0.82 (0.73, 0.92),** I^2^=77% (SoE: Moderate)  - Home-visit interventions (study=1, outcome: >30-day mortality): 0.52 (0.24, 1.1)  - Educational interventions (studies=2; outcome: >30-day mortality): 0.77 (0.38, 1.54)  - Clinic-based interventions (studies=1; outcome: >30-day mortality): **0.49 (0.3, 0.79)**  - Telemonitoring (studies=2; outcome: 30-day mortality): **0.64 (0.42, 1)**  - Telemonitoring (studies=2; outcome: >30-day mortality): 0.98 (0.81, 1.18)  - Telephone-based interventions (studies=3; outcome: >30-day mortality): 0.85 (0.6, 1.22)  - Multicomponent intervention (studies=1; outcome: 30-day mortality): 0.61 (0.3, 1.24)  - Multicomponent intervention (studies=1; outcome: >30-day mortality): 0.7 (0.42, 1.17)  Total: **0.79 (0.67, 0.93),** I^2^=40%; SoE of evidence: Moderate  - Telemonitoring (studies=2; outcome: 30-day ED visits): **0.62 (0.42, 0.94)**  - Telemonitoring (studies=1; outcome: >30-day ED visits): 0.93(0.81, 1.08)  Total: **0.73 (0.53, 1),** I^2^=82% (SoE: Low)  - TCIs (all together, expressed in SMD & 95%CI) (studies=6, outcome: >30-day QoL after discharge): -0.18(-0.65, 0.29), I^2^=98% (SoE: Very Low)  Certainty assessment  - Hospital re-admission (n studies: 9), (RR, 95%CI): 0.82(0.73, 0.92); certainty: Moderate  - Mortality (n studies: 10), (RR, 95%CI): 0.79(0.67, 0.93); certainty: Moderate  - ED visit (n studies:2), (RR, 95%CI): 0.73(0.53, 1), certainty: Low  - QoL (n studies:5), (SMD, 95%CI): -0.18(-0.65, 0.29), certainty: Very Low |
| Backman, 2020 | Canada; N=28; Relevant: 21 (Australia: 3, Italy: 1, Slovenia: 1, the Netherlands: 2, USA: 14); 4 databases; Inception – 11/2016 | SR | To assess the effects of PPFC on the quality of care and patients’ experiences during their transition from hospital to home. | Interventions: PFC-based interventions aiming to care transition (e.g.., nurse-initiated telephone follow-up, post-discharge telephone support, goal setting with CHWs, empowerment/educational sessions, in-hospital cognitive-based interventions)  Population: Adult cardiac patients | - QoL was assessed in 11 studies. 5/11 found a significant improvement in QoL of those attending a PFCC-based intervention (p<0.05). |
| Birtwell, 2022 | UK; N=19; Relevant: 14 (Australia, Denmark, USA); 5 databases; Inception 07/2021 | SR & MA (RCTs, cluster RCTs, non-randomised trials, pre- & post-intervention design) | To assess the effects of TCIs on patient-related outcomes transitioning into and out of LTCFs. | Interventions: TCIs (e.g., early assessment post-discharge interventions, acute nursing support, pharmacist transition coordinated interventions, outreach & nurse-led telephone triage lines, education & care plans’ implementation, multi-disciplinary teams in care management)  Population: LTCFs residents in transition from hospitals to LTFCs or from LTFCs to hospitals. | Meta-analysis results (OR, 95%CI)  - TCIs (studies=11; outcome: reduction in readmissions to hospitals): **1.48 (1.01, 2.17),** I^2^=40%  - TCIs (studies=5; outcome: reduction in readmissions ED): 2.04 (0.96, 4.33), I^2^ = 93%  - TCIs (studies=14; outcome: reduction in hospital & ED readmissions combined): **1.66 (1.18, 2.35)**, I^2^ = 81%  Meta-analysis results (SMD, 95%CI)  - TCIs (studies=3; outcome: LOS in ED): **−3.00 (−3.61, −2.39)**, I^2^ = 99%  - TCIs (studies=7; outcome: LOS in hospital): −1.86 (−5.47, 1.75), I^2^ = 98%  - TCIs (studies=2; outcome: QoL): −0.04 (−0.46 to 0.38), I^2^ = 92%  Meta-analysis results (RR, 95%CI)  - TCIs (studies=6; outcome: all-cause mortality): 0.95 (0.79, 1.16), I^2^= 0% |
| Chartrand, 2023 (linked to Backman, 2020) | Canada; N = 50; Relevant: 32 (Australia: 4, Canada: 1, Denmark: 4, Italy: 1, Norway: 1, Slovenia: 1, Spain: 1, Sweden: 1, the Netherlands: 1, USA:17); 3 databases; NR – 03/2021 | SR & MA (cluster/pilot-RCTs) | To assess the effects of PFC transition interventions on hospital readmissions and ED visits after discharge. | Interventions: Care transition interventions (e.g., discharge planning, needs assessment, medication reconciliation, telephone follow-up, home visits, and patient/caregiver education) based on PCC framework (i.e., holistic, collaborative, and responsive care)  Population: Adult patients at care transitions (hospital to home). | Meta-analysis results (OR, 95%CI)  - PFC interventions (studies= 10; outcome: number of patients readmitted ≥ 1 times): 0.93 (0.74, 1.18), I^2^=46%  - PFC interventions (studies= 4; outcome: number of patients readmitted at least once 1–8 years after discharge): **0.63, (0.44, 0.91)**, I^2^=32%  - PFC interventions (studies= 3; outcome: number of patients who visited the ED after discharge ≥ 1 times): **0.56 (0.34, 0.95)**, I^2^=51%  Meta-analysis results (IRR, 95%CI)  - PFC interventions (studies= 24; outcome: number of incidents of hospital readmission): **0.86 (0.75, 0.98)**, I^2^=73%  - PFC interventions (studies= 5; outcome: number of incidents of ED visits): 1.0 (0.85, 1.18), I^2^= 29% |
| Chang, 2023 | USA; N=31; most of studies conducted in USA; 8 databases; 01/2000 – 03/2021 | SR & MA (RCTs & observational studies) | To assess the effects of interventions targeting HNHC patients | Interventions: Home-Based Care; Primary Care–Based; Ambulatory Intensive Caring Unit–Based; ED-based; Community-based; Telephonic/mail; System-Level Transformation  Population: HNHC patients (mostly adults) | Home-based care interventions  - reductions in hospitalizations for ACSC  - no changes in mortality (SoE: Low)  Primary care interventions  - reductions in hospitalizations (SoE: Low)  - reductions in total costs (SoE: Low)  Ambulatory Intensive Caring Unit–Based Interventions  - Inconsistent findings for all outcomes (SoE: Insufficient)  Emergency Department–Based Interventions  - reductions in ED visits (SoE: Moderate)  - reductions in ED costs (SoE: Low)  - reductions in hospitalisations (SoE: Low)  - no changes in hospital costs (SoE: Low)  Community-Based Interventions  - No changes in mortality (SoE: Low)  - Rest of use and cost outcomes (SoE: Insufficient)  Telephonic/Mail Interventions  - No changes in the number of ED visits (SoE: Low)  - No changes in the number of ACSC ED visits (SoE: Low)  - No changes in the ACSC hospital admissions (SoE: Low)  - No changes in total healthcare costs (SoE: Low)  - No changes in mortality rate (SoE: Low)  Meta-analysis results (MD, 95%CI)  System-Level transformation (n studies= 5; outcome: total annual costs): −$13 (−132, 106), I^2^=91%; (SoE: Low)  Primary care-based (n studies=3; outcome: total annual costs): **−$4119 (−7935, -302)**, I^2^=29%; (SoE: Low)  Telephonic/mail interventions (n studies=3; outcome: total annual costs): $837 (−7428, 9109), I^2^=46%; (SoE: Low) |
| Chauhan, 2022 | Canada; N = 24; Relevant: 18 (Australia; Belgium; Canada; Denmark; the Netherlands; Spain; Switzerland; USA); 2 databases; 01/2000 – 06/2001 | SR & MA (RCTs) | To assess the effects of VW interventions on patient-related outcomes at post-discharge. | Interventions: Post-discharge VW in the community (e.g., home-visits, care coordination, and daily case management with a multidisciplinary)  Population: Adult patients at post-discharge | Meta-analysis results (RR, 95%CI)  - VWs (studies= 11; outcome: hospital readmission in HF patients): **0.84 (0.74, 0.96)**, I^2^ = 59%  - VWs (studies= 3; outcome: hospital readmission in COPD patients): 0.97 (0.62, 1.51), I^2^ = 75%  - VWs (studies= 3; outcome: hospital readmission in patients at high-risk of readmission): 1.00 (0.95, 1.06), I^2^ = 0%  - VWs (studies= 5; outcome: hospital readmission in patients with mixed diagnosis): 0.91 (0.73, 1.14), I^2^ = 74%  Total: **0.91 (0.85, 0.98)**, I^2^ = 60%  - VWs (studies= 3; outcome: ED visits in HF patients): 0.65 (0.39, 1.11), I^2^ = 82%  - VWs (studies= 2; outcome: ED visits in COPD patients): 0.67 (0.17, 2.57), I^2^ = 64%  - VWs (studies= 2; outcome: ED visits in patients at high-risk of readmission): 0.91 (0.68, 1.22), I^2^ = 71%  - VWs (studies= 3; outcome: ED visits in patients with mixed diagnosis): 0.85 (0.55, 1.33), I^2^ = 64%  Total: **0.83 (0.70, 0.98)**, I^2^ = 69%  - VWs (studies= 10; outcome: mortality in HF patients): **0.86 (0.76, 0.97)**, I^2^ = 0%  - VWs (studies= 3; outcome: mortality in COPD patients): 1.11 (0.69, 1.79), I^2^ = 0%  - VWs (studies= 2; outcome: mortality in patients at high-risk of readmission): 0.97 (0.84, 1.12), I^2^ = 0%  - VWs (studies= 4; outcome: mortality in patients with mixed diagnosis): 0.84 (0.68, 1.02), I^2^ = 0%  Total: **0.90 (0.82, 0.97)**, I^2^ = 0%  Meta-analysis results (MD, 95%CI)  - VWs (studies= 4; outcome: LOS for readmission following discharge): **–1.94 (–3.28, –0.60)**, I^2^ = 8%  Meta-analysis results (SMD, 95%CI)  - VWs (studies= 6; outcome: QoL): 0.11 (−0.01, 0.24) |
| Hobohm, 2023 | Germany; N=22; Relevant: 21 (Canada: 1; Poland: 1; USA: 18); 3 databases; NR – 01/2022 | Scoping review & MA (Prospective & retrospective observational studies) | To assess the impact of PERTs in acute PE treatment. | Interventions: PERTs  Population: Patients with PE | - Mortality rate (n studies=11) (%, n/N, 95%CI): 10% [177/1532 patients (8, 13%)]  - LOS (n studies=11) (mean days, 95%CI): 7.3 (5.7, 8.9)  - No difference in 30-day readmission  - No difference in ICU admission rates  PERT era versus pre-PERT era (n studies=9)  - Mortality (RR, 95%CI): 0.89 (0.67, 1.19)  - Mortality (subgroups: patients with intermediate/high-risk PE): 0.71 (0.45, 1.12)  - LOS in hospital (in days) (MD, 95%CI): **-1.61 (-3.21, -0.02)** (PERT versus pre-PERT era), I^2^>90%  - LOS in ICU (in days) (MD, 95%CI): **-1.79 (-3.29, -0.28),** I^2^>90% |
| James, 2023 | Canada; N=31; Relevant: USA (n=26); Canada, France and Denmark; 3 databases; 2010 – 08/2022 | SR | To assess the impact of interventions at pre- and during the transition from hospital to community. | Interventions: Pre- and during the transitions from hospital to community (including pharmacotherapy).  Population: individuals with substance-use disorders | Hospital readmission in months (12 studies): 10/12 studies found a significant reduction in hospital readmissions from 1-12 months post-discharge for those at intervention groups.  ED visits (in months) (5 studies): 4/5 studies found a significant reduction in ED revisits from 1-12 months post-discharge for those at intervention groups. |
| Leduc, 2021 | Canada; N=22; Relevant: 22 (Canada: 5; Norway: 1; Scotland: 1; USA: 15); 3 databases; NR – 02/2019 | SR | To assess the effectiveness of “on-site” interventions in reducing the volume of ED visits | Intervention: Advanced nursing care (e.g., nurse practitioners); programs that used a set of tools called INTERACT; primary interventions plus INTERACT  Population: Long-term care patients | - 14/16 interventions were found effective in decreasing the transfer of patients to the hospital with this decrease ranging from 5-69% (statistically significant estimates were found in seven interventions)  - 20/21 interventions were found effective to decrease hospitalisations (statistically significant effects were found in seven interventions)  - Mortality (n studies=5): slight decrease (non-significant)  - LOS (n studies=3) (range): 0.2 to 1.2 days fewer  - Cost impacts (n studies=3) (average saving per patient): $512 |
| Li, Li, 2021 | China; N=25; Relevant: 21 (Canada: 2; Germany: 1; Ireland: 1; Italy: 1; Spain: 1; Sweden: 2; UK: 1; USA: 12); 4 databases; 01/2000 – 06/2020 | SR & MA (RCTs) | To assess the effects of nurse-led TCIs on readmissions, ED visits, LOS. | Interventions: Nurse-led TCIs  Population: HF patients | - Nurse-led TCIs versus usual care (studies= 19; outcome: All-cause readmissions) (RR, 95%CI): **0.91 (0.82, 0.99),** I^2^=49%  - Nurse-led TCIs versus usual care (n studies= 10; outcome: HF-specific readmissions) (RR, 95%CI): **0.71 (0.6, 0.84),** I^2^=0%  - Nurse-led TCIs versus usual care (n studies= 5; outcome: ED-visits) (RR, 95%CI): 0.96 (0.84, 1.10), I^2^=0%  - Nurse-led TCIs versus usual care (n studies=5; outcome: hospital LOS) (MD, 95%CI): **-2.37 (-3.16, -1.58),** I^2^=14% |
| Li, Fu, 2021 | China; Relevant: 30 (USA: 15; rest: Germany, Spain, Belgium, Sweden, Ireland, UK, Australia and Canada); N=38; 4 databases; 01/2009 – 10/2019 | SR & MA (RCTs) | To assess the effects of nurse-led TCIs on readmissions, ED visits, LOS in patients hospitalised for HF. | Intervention: Nurse-led TCIs  Population: HF patients discharged from hospital | - Primarily educational interventions (studies= 2; outcome: all-cause readmissions) (RR, 95%CI): 0.92(0.71, 1.18), I^2^=0%  - Home-visiting programmes (studies= 4; outcome: all-cause readmissions) (RR, 95%CI): 0.91(0.74, 1.13), I^2^=26%  - Structured telephone support (studies= 5; outcome: All-cause readmissions) (RR, 95%CI): 0.91(0.72, 1.14), I^2^=67%  - case management (studies= 10; outcome: All-cause readmissions) (RR, 95%CI): 0.96(0.85, 1.09), I^2^=44%  - telemonitoring (studies= 2; outcome: All-cause readmissions) (RR, 95%CI): 1.24(0.83, 1.86), I^2^=11%  - clinic-based intervention (studies= 1; outcome: All-cause readmissions) (RR, 95%CI): **0.51(0.29, 0.91)**  - multi-disciplinary care model (studies= 3; outcome: All-cause readmissions) (RR, 95%CI): **0.66 (0.55, 0.79)**, I^2^=0%  - peer-support intervention (studies= 1; outcome: All-cause readmissions) (RR, 95%CI): 0.88 (0.45, 1.71)  - short-message service (studies= 1; outcome: All-cause readmissions) (RR, 95%CI): **0.79 (0.63, 0.99)**  Total: **0.89 (0.82, 0.97)**, I^2^=54%  - primarily educational intervention (studies=1; outcome: HF-specific readmissions) (RR, 95%CI): 0.97(0.5, 1.87)  - home-visiting programmes (studies=3; outcome: HF-specific readmissions) (RR, 95%CI): 0.87(0.6, 1.25), I^2^=63%  - structured telephone support (studies=5; outcome: HF-specific readmissions) (RR, 95%CI): **0.72(0.58, 0.89)**, I^2^=0%  - case management (studies=2; outcome: HF-specific readmissions) (RR, 95%CI): 0.68(0.42, 1.12), I^2^=57%  - telemonitoring (studies=1; outcome: HF-specific readmissions) (RR, 95%CI): 1.7(0.82, 3.51)  - clinic-based intervention (studies=1; outcome: HF-specific readmissions) (RR, 95%CI): 0.71(0.47, 1.09)  - multidisciplinary care model (studies=2; outcome: HF-specific readmissions) (RR, 95%CI): **0.37(0.17, 0.8)**, I^2^=75%  - peer-support intervention (studies=1; outcome: HF-specific readmissions) (RR, 95%CI): 1.91(0.62, 5.89)  - short-message service (studies=1; outcome: HF-specific readmissions) (RR, 95%CI): 0.84(0.62, 1.16)  Total: **0.78 (0.68, 0.89)**, I^2^=33%  - home-visiting programmes (studies= 1; outcome: ED visits) (RR, 95%CI): 1.12(0.77, 1.61)  - structured telephone support (studies= 2; outcome: ED visits) (RR, 95%CI): 0.61(0.21, 1.77), I^2^=0%  - case management (studies= 2; outcome: ED visits) (RR, 95%CI): 0.9(0.75, 1.09), I^2^=0%  - multidisciplinary care model (n studies= 1; outcome: ED visits) (RR, 95%CI): 0.96(0.78, 1.18)  Total: 0.94 (0.83, 1.07), I^2^=0%  - Total hospital LOS was assessed in four studies. 3/4 showed reduced LOS favouring the nurse-led TCIs. LOS per patient was assessed in eight studies with none of them finding statistically significant effects. |
| Osterlind, 2020 | Sweden; N=15; Relevant: 11 (Australia, Canada, New Zealand, UK); 5 databases; Inception – 02/2020 | SR & MA (pre- and post-intervention cohort studies) | To assess the effects of critical care transition programmes on ICU readmission & in-hospital mortality. | Interventions: CCTPs (e.g., critical care/nursing outreach service, rapid response team, ICU consult service, medical emergency team, ICU liaison nurse).  Population: Adult ICU discharged patients | Meta-analysis results (RR, 95%CI)  - CCOS (studies = 5; outcome: ICU readmission): **0.64 (0.42, 0.99)**, I^2^ = 81%  - ICU liaison nurse (studies= 2; outcome: ICU readmission): 0.96 (0.67, 1.38), I^2^ = 0%  - RDP (studies = 1; outcome: ICU readmission): 1.02 (0.53, 1.95)  - RRT (studies = 2; outcome: ICU readmission): **0.76 (0.67, 0.87)**, I^2^ = 0%  - MET (studies=1; outcome: ICU readmission): 1.56 (0.78, 3.1)  Total: **0.78 (0.64, 0.96)**, I^2^ = 63% (SoE: Very low)  - CCOS (studies = 5; outcome: in-hospital mortality after ICU): **0.62 (0.43, 0.9)**, I^2^ = 87%  - ICU liaison nurse (studies= 2; outcome: in-hospital mortality after ICU): 0.69 (0.23, 2.09), I^2^ = 58%  - RDP (studies = 1; outcome: in-hospital mortality after ICU): 0.99 (0.62, 1.56)  - RRT (studies = 2; outcome: in-hospital mortality after ICU): 1.30 (0.54, 3.16), I^2^ = 95%  - MET (studies=1; outcome: in-hospital mortality after ICU): 1.17 (0.84, 1.62)  Total: 0.82 (0.64, 1.06), I^2^ = 87% (SoE: Very low) |
| Rush, 2020 | Canada; N = 14; Relevant: 12 (Australia, Canada, Italy, Spain, USA); 4 databases; Inception – 05/2019 | SR | To assess the impact of TCIs on mortality, QoL, ED visits, and hospitalisations. | Interventions: TCIs from in-hospital to home/community or from ED to community/home (e.g., pathways/algorithms, discharge and/or post-discharge education in-person/telephone follow-up at home or at a clinic, use of care guidelines/ multidisciplinary disease management)  Population: Atrial fibrillation patients | Outcome (QoL; 3 studies): Statistically significant improvements in QoL for those attending TCIs (clinical pathways with atrial fibrillation clinic referral/follow-up or management education applications) from ED and hospital to outpatient community care.  Outcome (Mortality; 4 studies): Mixed & inconsistent findings.  Outcome (Hospital readmissions; 6 studies): Mixed & inconsistent findings.  Outcome (Hospital LOS; 1 study): Statistically significant improvements for those patients admitted to a clinical pathway from ED to cardiology services.  Outcome (Costs; 1 study): Implementation of an ED-based observation unit involving a practice algorithm was found to be cost-effective compared to hospitalisation. |
| Saunders, 2019 | Canada; N=8; Relevant: 5 (USA); 4 databases; 1995 – 04/2018 | SR | To assess the impact of inpatient specialised palliative care programmes on patients’ transition from hospital to the community setting. | Intervention: any inpatient specialist palliative care intervention delivered by palliative care specialists healthcare professionals (i.e., screening tools, discharge planning to the community, interventions that spanned hospital and community).  Population: Adults patients with progressive/serious illnesses or patients with life expectancy ≤ than 2 years. | Outcome (Hospital LOS; 4 studies): One study found no significant changes between the intervention and UC groups. One study found that patients in the intervention group had a statistically significant longer median LOS. Two studies found that those in the intervention groups had a short hospital stay, although the effects were not statistically significant.  Outcome (QoL; 1 study): No significant differences between the intervention and UC group (baseline period) were detected.  Outcome (Mortality; 1 study): No significant differences between the intervention and UC groups (baseline period) were detected.  Outcome (Hospital readmissions; 4 studies): Two studies found statistically significant improvements favouring the intervention groups compared to the baseline period. The rest two studies found no statistically significant effects. |
| Sezgin, 2020 | Ireland; N=113 (total), with most of them (n=75) conducted in the USA; 4 databases; 01/2002 – 02/2019 | Scoping review | To assess the effectiveness of intermediate care interventions (including TCIs) in reducing hospital LOS, ED admissions, health, and social care costs. | Interventions: Intermediate care interventions (including TCIs)  Population: middle-aged and older adults (aged ≥ 50) | - TCIs delivered exclusively in hospital (n studies=8): reduced readmission was detected was associated with coaching interventions delivered by social workers, hospitalists/nurses. No reduction in readmissions was associated with interventions combining patients’ education and discharge planning, however reduced rates of admission at long-term were observed.  - Outreach by hospital professionals (combined interventions including education, assessment, follow-up, medication management) (n studies=51): Outreach interventions delivered by interdisciplinary teams were related with reduced hospital stay.  - Community based or in‑reach models (n studies = 24): These models were related with reduced hospital stay, fewer readmissions and lower costs. Almost all those models were consisted of combined interventions.  - Transitional care clinics combined with telephone and/or nurse follow up (n studies = 7): A trend of reduced rehospitalisation was observed for these interventions.  - Telephone follow‑up (n studies = 69): Inconsistent results regarding their effects on discharge from ED, 30-days rehospitalisation and mortality. Inconsistent results for the interventions delivered by different healthcare professionals.  - Patient and caregiver education or coaching interventions (n studies= 65): Generally effective in relation to costs and readmission and QoL.  - Decision support systems (electronic health records & patient information tools) (n studies= 5): Inconsistent findings on readmissions.  - Interdisciplinary support with rehabilitation (most were multidisciplinary geriatrician/physician-led programmes) (n studies = 19): Some evidence of reducing mean LOS in hospitals.  - Single profession-led interventions (n studies= 10): Generally effective in reducing hospital readmissions.  - Crisis response or hospital admission avoidance (n studies= 7): Generally, not cost-effective.  - Assessment and rehabilitation (most were delivered on site or by telephone advice by interdisciplinary teams led by physicians), (n studies= 11): Generally, effective in reducing hospital costs and hospital LOS. |
| Tanner, 2021 | UK; N = 8; Relevant: 6 (Australia, Canada, UK, USA); 6 databases; NR - 2019 | SR & MA (Pre- & post-intervention cohort studies) | To assess the impact of routine CCSDPs readmission and/or mortality  in patients discharged from critical care to general wards | Intervention: CCDPs (CCSDP-contained nurses)  Population: Adult critical care patients | Meta-analysis results (RR, 95%CI)  - CCDPs (studies=4; outcome: Readmission to critical care): 0.85 (0.66, 1.09), I^2^ = 63%  - CCDPs (studies=2; outcome: Readmission to critical care within 72 hours): **1.49**  **(1.05, 2.12)**, I^2^ = 0% (favouring the pre-intervention group)  - CCDPs (studies=4; outcome: mortality following critical care discharge): 0.90 (0.75, 1.07), I^2^ = 40% |
| Totten, 2019 | USA; N=233; Relevant: (Australia/New Zealand: 19, Canada: 4, UK: 22, USA: 110); 3 databases; 1996 – 05/2018 | SR & MA (RCTs, cohorts, pre- and post-intervention designs) | To assess the effects of telehealth consultations on intermediate care outcomes, costs, QoL. | Interventions: Inpatient telehealth consultations  Population: Patients (no restrictions applied) | - Remote ICU via inpatient telehealth consultations is associated with lower hospital mortality (SoE: Moderate); inconsistent effects on cost-effectiveness (SoE: Insufficient); not statistically significant effects on LOS in ICU and hospitals (SoE: Moderate).  - Inpatient Specialist telehealth Consultations are associated with reduced mortality (not statistically significant) (SoE: Low); no effects on cost-effectiveness (SoE: Low); reduced hospital LOS (not statistically significant) (SoE: Low).  - Tele-stroke services not effective in 3-month mortality (SoE: Moderate); Specialty consultations in ED showed generally positive effects on mortality (not statistically significant results) (SoE: Low), generally positive effects on waiting times in ED (SoE: Moderate) and savings (SoE: Low).  - Outpatient telehealth consultations generally reduce hospital LOS, number of hospitalisations and hospital visits.  - Meta-analysis results (RR, 95%CI) (n studies: 11; outcome: ICU mortality): **0.69 (0.51, 0.89)**, I^2^=79.6%  - Meta-analysis results (RR, 95%CI) (n studies: 11; outcome: hospital mortality in ICU studies): **0.76 (0.6, 0.95)**, I^2^=79.4%  - Meta-analysis results (MD, 95%CI) (n studies: 12; outcome: ICU LOS): -0.39 (-0.99, 0.15), I^2^=91%  - Meta-analysis results (MD, 95%CI) (n studies: 12; outcome: LOS in ICU studies): -0.14 (-0.96, 0.63), I^2^=92.8%  - Meta-analysis results (RR, 95%CI) (n studies: 18 studies; intervention: telestroke; outcome: in-hospital mortality): 0.64 (0.22, 1.41), I^2^=0% |
| Tyler, 2023 | UK; N=126; Relevant: 88 (conducted in OECD countries); 5 databases; Inception – 08/2022 | SR (with NMA & MA) (RCTs plus cluster) | To investigate the comparative effectiveness of TCIs on healthcare utilisation and patient-at transition related outcomes. | Interventions: TCIs implemented at pre-discharge/during discharge/after-discharge from hospital to community.  Population: All patients irrespective of age. | Network meta-analysis results (OR, 95%CI)  - Low-complexity versus UC (studies=73; outcome: 30-day hospital readmissions): **0.78 (0.66, 0.92)**  - Medium-complexity versus UC (studies=73; outcome: 30-day hospital readmissions): **0.82 (0.68, 0.97)**  - High-intensity versus UC (studies=73; outcome: 30-day hospital readmissions): 0.96 (0.80, 1.15)  Head-to-head comparisons compared to minimal interventions (outcome: 30-day hospital readmissions).  - Low-complexity: **0.50 (0.32, 0.77)**  - Medium-complexity: **0.52 (0.33, 0.81)**  - High-complexity: **0.61 (0.40, 0.92)**  - Low-intensity versus UC (studies=34; outcome: 90-day hospital readmissions): 0.65 (0.41, 1.02)  - Medium-complexity versus UC (studies=34; outcome: 90-day hospital readmissions): **0.64 (0.45, 0.92)**  - High-complexity versus UC (studies=34; outcome: 90-day hospital readmissions): **0.72 (0.57, 0.91)**  - Low-complexity versus UC (studies=34; outcome: 180-day hospital readmissions): **0.45 (0.30, 0.66)**  - Medium-complexity versus UC (studies=34; outcome: 180-day hospital readmissions): **0.57 (0.35, 0.91)**  - High-complexity versus UC (studies=34; outcome: 180-day hospital readmissions): **0.78 (0.62, 0.98)**  - Low-complexity versus UC (studies=41; outcome: ED visits): **0.68 (0.48, 0.96)**  - Medium- and high-complexity: NS  Meta-analysis results (SMD, 95%CI)  - High-complexity versus UC (studies=12; outcome: LOS in hospital): **−0.20 (−0.38, −0.03),** I^2^=75%  - Low-complexity versus UC (studies=6; outcome: LOS in hospital): 0.11 (-0.42, 0.65), I^2^=84%  - Medium-complexity versus UC (studies=5; outcome: LOS in hospital): -0.1 (-0.39, 0.19), I^2^=43%  - High-complexity versus UC (studies=14; outcome: general QoL): 0.37 (-0.17, 0.91), I^2^=97%  - Low-complexity versus UC (studies=5; outcome: general QoL): 0.11 (-0.05, 0.27), I^2^=0%  - Medium-complexity versus UC (studies=7; outcome: general QoL): 0.1(-0.14, 0.34), I^2^=35% |
| van den Broek, 2023 [38] | The Netherlands; N=12; Relevant: 12 (Australia, Belgium, Canada, UK, USA); 5 databases; NR – 01/2023 | SR | To assess the impact of ED-based interventions | Interventions: ED-based interventions delivered by healthcare professionals specialised to transitional care (i.e., ED-nurse discharge service, nurse-led transitional care, care coordination team, patient navigator).  Population: older adults (aged over 65) | ED revisit (8 studies): 5/8 studies found a statistically significant effect of ED-based interventions in decreasing ED-visits. One study found statistically significant effects favouring the control/pre-intervention group. Two studies reported NS findings.  Hospital readmissions (6 studies): 5/6 studies found a statistically significant effect of ED-based interventions in reducing hospital readmissions. One study found statistically significant effects favouring the control/pre-intervention group.  LOS (ED/hospital) (3 studies): One study found a statistically significant effect of ED-based interventions in reducing LOS, one study found statistically significant effect of control/pre-intervention group in reducing LOS, and one study reported NS results. |
| Weeks, 2018 | Canada; N= 23; Relevant: 18 (Canada, Germany, Sweden, Switzerland, USA); 6 databases; Inception – 05/2016 | SR & MA (RCTs & case-control designs) | To assess the impact of TCIs on healthcare utilisation outcomes. | Interventions: TCIs (e.g., coordinating & facilitating care teams; health and community services; education; health monitoring; health management, physical/environmental assessments; medication support; supporting empowerment, autonomy, and self-management)  Population: community-dwelling adults | Meta-analysis results (OR, 95%CI)  - TCIs (studies= 10; outcome: 30-day hospital readmissions): **0.75 (0.62, 0.91)**, I^2^ = 52%  - TCIs (studies= 6; outcome: 90-day hospital readmissions): 0.77 (0.59, 1.02), I^2^ = 67%  - TCIs (studies= 6; outcome: 180-day hospital readmissions): **0.81 (0.72, 0.92)**, I^2^ = 86%  ED-visits (5 studies): One study (1/5) found statistically significant effects of TCIs on ED-visits. |
| Wong, 2021 | Hong Kong; N=12; Relevant: 10 (Australia, Canada, UK, USA); 5 databases; Inception – 08/2019 | SR (with NMA & MA) (RCTs) | To investigate the comparative effectiveness nurse-led peri-discharge interventions compared to UC on all-cause 30-day hospital readmissions. | Interventions: interventions (NLPD) (no restrictions applied).  Population: Adult patients (aged over 18 years) who were admitted from the community. | Meta-analysis results (RR, 95%CI)  - NLPD interventions (studies=12; outcome: all-cause 30-day hospital readmission): 0.86 (0.71, 1.04) (SoE: Moderate)  - NLPD interventions (studies=3; outcome: all-cause 30-day mortality): 0.26 (0.05, 1.32) (SoE: Moderate)  - NLPD interventions (studies=5; outcome: all-cause 30-day ED-visits): 0.96 (0.82, 1.12) (SoE: High)  NMA findings: NS differences among the 16 reported interventions. |
| ***Note.*** ACSC: ambulatory-care sensitive conditions; CCDPs: critical care stepdown programmes; CCOS: Critical Care outreach service; CCTPs: Critical care transition programmes; CG: control group; CHW: community health workers; CI: confidence intervals; COPD: Chronic obstructive pulmonary disease; ED: emergency department; HF: heart failure; HNHC: high-need, high-cost; ICU: intensive care unit; INTERACT: Interventions to Reduce Acute Care Transfers; IRR: incidence rate ratio; LOS: length of stay; LTCFs: long-term care facilities; MA: meta-analysis; MET: Medical emergency team; MD: mean difference; NLPD: nurse-led peri-discharge; NMA: Network meta-analysis; NR: Not reported; NS: not statistically significant; OECD: Organisation for Economic Co-operation and Development; PCC: patient-centred care; PE: pulmonary embolism; PERTs: pulmonary embolism response teams; PFC: Person- and family-centred care; QoL: quality of life; RDP: redesigned discharge process; RR: risk-ratio; RRT: Rapid response team; SMD: standardised mean difference; SoE: strength of evidence; SR: systematic review; TCIs: transitional care interventions; UC: usual care; VW: Virtual ward.  *Statistically significant estimates in meta-analyses (p≤0.05) are reported in bold (where available).  **Non-relevant studies (conducted in non-Westernised countries) may be included in the reported meta-analysis estimates. | | | | | |

**Appendix Figure 1.** Heatmap showing the CCA (%) for pairs of included reviews (the grey diagonal tiles present the single/ total number of primary studies that are included in each review).


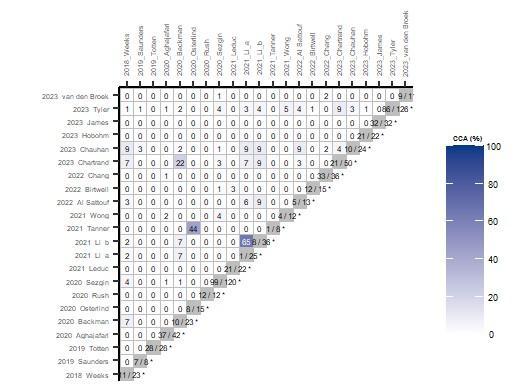


**Appendix Figures 2-3**: Critical appraisal of the included reviews (confidence ratings graph and table with the critical appraisal ratings by study).


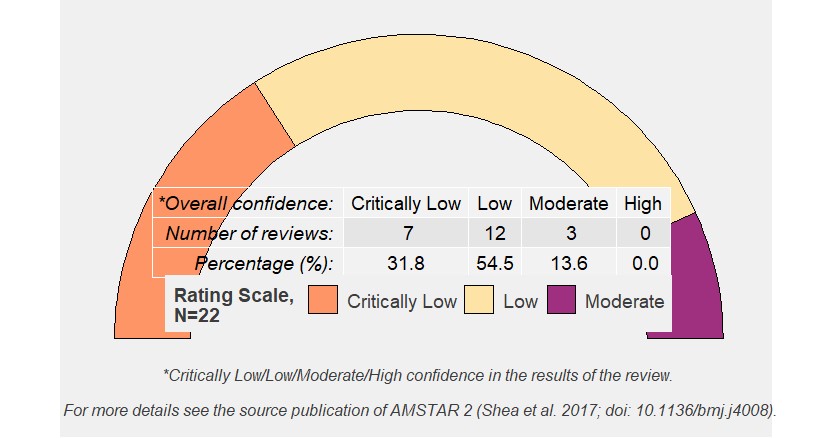


|  | AMSTAR 2 items | | | | | | | | | | | | | | | |  |
| --- | --- | --- | --- | --- | --- | --- | --- | --- | --- | --- | --- | --- | --- | --- | --- | --- | --- |
| Reviews | 1. PICO components*^A^* | 2.* Pre-established protocol*^B^* | 3. Explanation of included studies' design*^A^* | 4.* Comprehensive search strategy*^B^* | 5. Duplicate study selection*^A^* | 6. Duplicate data extraction*^A^* | 7.* List of excluded studies and justification*^B^* | 8. Description of included studies*^B^* | 9.* Assessment of RoB in included studies*^B,C^* | 10. Funding sources*^A^* | 11.* Use of appropriate statistical methods*^C,D^* | 12. RoB impact on synthesized results*^D^* | 13.* Results interpretation with RoB reference*^A^* | 14. Heterogeneity explanation*^A^* | 15.* Publication/ small study bias investigation*^D^* | 16. Conflict of interest declaration*^A^* | Overall confidence*^E^* |
| Aghajafari_  2020 | Yes | Yes | Yes | Yes | Yes | Yes | No | Yes | Yes | Yes | Yes | No | Yes | Yes | Yes | Yes | Low |
| Al Sattouf_2022 | Yes | No | Yes | Partial Yes | No | No | No | Partial Yes | Partial Yes | No | Yes | Yes | No | Yes | No | Yes | Critically Low |
| Backman_  2020 | Yes | Yes | No | Partial Yes | Yes | Yes | Partial Yes | Partial Yes | Partial Yes | No | No MA | No MA | Yes | NA | No MA | Yes | Moderate |
| Birtwell_  2022 | Yes | Yes | Yes | Yes | Yes | No | Yes | Yes | Partial Yes | No | Yes | Yes | Yes | Yes | Yes | Yes | Moderate |
| Chang_2022 | Yes | Yes | Yes | Partial Yes | Yes | No | No | Partial Yes | Partial Yes | No | Yes | No | Yes | Yes | No | Yes | Critically Low |
| Chartrand_  2023 | Yes | Yes | Yes | Partial Yes | Yes | Yes | Partial Yes | Partial Yes | Partial Yes | No | Yes | No | Yes | Yes | No | Yes | Low |
| Chauhan_  2023 | Yes | No | No | Partial Yes | Yes | No | No | Yes | Partial Yes | No | Yes | No | Yes | No | No | Yes | Critically Low |
| Hobohm_  2023 | Yes | Yes | Yes | Partial Yes | Yes | Yes | No | Yes | Partial Yes | No | Yes | No | Yes | No | Yes | Yes | Low |
| James_2023 | Yes | No | No | Partial Yes | No | No | No | Partial Yes | Partial Yes | No | No MA | No MA | Yes | NA | No MA | Yes | Critically Low |
| Leduc_2021 | Yes | Yes | No | Partial Yes | Yes | No | No | Yes | Partial Yes | No | No MA | No MA | Yes | NA | No MA | Yes | Low |
| Li, Li_2021 | Yes | Yes | Yes | Partial Yes | Yes | Yes | No | Yes | Yes | No | Yes | Yes | Yes | Yes | Yes | Yes | Low |
| Li, Fu_2021 | Yes | Yes | Yes | Partial Yes | Yes | Yes | No | Yes | Yes | No | Yes | Yes | Yes | Yes | Yes | No | Low |
| Osterlind_  2020 | Yes | Yes | Yes | Partial Yes | Yes | Yes | No | Partial Yes | Partial Yes | No | Yes | Yes | Yes | Yes | Yes | Yes | Low |
| Rush_2020 | Yes | No | No | Partial Yes | No | No | No | Partial Yes | Partial Yes | No | No MA | No MA | Yes | NA | No MA | Yes | Critically Low |
| Saunders_  2019 | Yes | Yes | No | Partial Yes | Yes | No | No | Partial Yes | Partial Yes | No | No MA | No MA | Yes | NA | No MA | Yes | Low |
| Sezgin_  2020 | Yes | Yes | Yes | Partial Yes | Yes | No | No | Partial Yes | No | No | No MA | No MA | No | NA | No MA | Yes | Critically Low |
| Tanner_  2021 | Yes | Yes | Yes | Partial Yes | No | No | Yes | Partial Yes | Partial Yes | No | Yes | Yes | Yes | Yes | Yes | No | Moderate |
| Totten_  2019 | Yes | Yes | Yes | Yes | Yes | No | Yes | Yes | Partial Yes | Yes | Yes | Yes | Yes | Yes | No | Yes | Low |
| Tyler_2023 | Yes | Yes | Yes | Partial Yes | Yes | No | No | Partial Yes | Yes | No | Yes | Yes | Yes | Yes | Yes | Yes | Low |
| van den Broek_  2023 | Yes | Yes | Yes | Partial Yes | Yes | Yes | No | Partial Yes | Partial Yes | No | No MA | No MA | Yes | NA | No MA | Yes | Low |
| Weeks_  2018 | Yes | No | No | Partial Yes | Yes | Yes | Yes | Partial Yes | Partial Yes | No | Yes | No | Yes | Yes | No | Yes | Critically Low |
| Wong_  2021 | Yes | Yes | Yes | Partial Yes | Yes | No | No | Partial Yes | Partial Yes | No | Yes | Yes | Yes | No | Yes | Yes | Low |
| *Asterisk indicates a critical item (domain). | | | | | | | | | | | | | | | | | |

**Appendix Table 11.** Risk-of-bias assessment (overall rating) of primary studies included in the meta-analytic reviews of this overview.

| **Study ID** | **RoB (overall)** |
| --- | --- |
| 2007_Afilalo | H |
| 2006_Baren | H |
| 2005_Basic | H |
| 1995_Batel | H |
| 2016_Bell | L |
| 2014_Biese | H |
| 2006_Brown | H |
| 2004_Caplan | H |
| 2015_Cossette_a | L |
| 2010_Currier | H |
| 2016_Edgren | H |
| 2017_Eisenstein | U |
| 2015_Griffey | L |
| 2015_Harvard | U |
| 2012_Jesudason | L |
| 1987_Jones | H |
| 1990_Jones | H |
| 2007_Kolbasovsky | H |
| 2005_Kyriacou | H |
| 2007_Lee | U |
| 2003_Lightbody | H |
| 2003_McCusker_a | U |
| 2003_Mion | L |
| 2016_Neven | H |
| 2011_Patel | H |
| 2016_Rathlev | U |
| 2007_Richards | L |
| 2000_Ritchie | H |
| 2013_Rosted | H |
| 1996_Runciman | H |
| 2015_Sharp | L |
| 1998_Spooren | H |
| 2017_Stergiopoulos | H |
| 1996_Townsend | H |
| 2006_Vaiva | H |
| 2015_Wexler | L |
| 1991_Zeiger | H |
| 2004_Wong | U |
| 2011_Yim | H |
| 2021_Dawson | SOME CONCERNS |
| 2021_Kazemi | L |
| 2020_Deek | SOME CONCERNS |
| 2020_You | SOME CONCERNS |
| 2018_Frederix | L |
| 2017_Boyde | SOME CONCERNS |
| 2014_Gonsalez | SOME CONCERNS |
| 2015_Yu | SOME CONCERNS |
| 2015_Vinluan | SOME CONCERNS |
| 2018_Cordato | H |
| 2011_Crilly | H |
| 2004_Crotty | H |
| 2005_Crotty | U |
| 2012_Elliott | H |
| 2014_Harvey | H |
| 2016_Hullick | H |
| 2017_Kane | H |
| 2020_Layton | H |
| 2002_Lee | H |
| 2012_Mudge | H |
| 2016_Mukamel | H |
| 2018_Pedersen | H |
| 2019_Shrapnel | H |
| 2015_Street | H |
| 2019_Aboumatar | H |
| 2018_Al-Hashar | H |
| 2000_Andersen | U |
| 2015_Bronstein | U |
| 2018_Collinsworth | H |
| 2019_Cui | H |
| 2018_Fors | U |
| 2009_Hanssen | H |
| 2021_Henschen | H |
| 2020_Hu | U |
| 2005_Huang | U |
| 2021_Kazemi Majd | L |
| 2019_Lembeck | H |
| 2021_Liang | H |
| 2019_Lindhardt | H |
| 2019_Lisby | H |
| 2006_Lopez Cabezas | H |
| 2021_Magny-Normilus | H |
| 1994_Naylor | H |
| 2018_Nguyen | H |
| 2014_Oliveira-Filho | U |
| 2006_Pearson | H |
| 2020_Piette_a | U |
| 2020_Piette_b | H |
| 1993_Schneider | H |
| 2021_Schnipper | H |
| 2018_Shahrokhi | U |
| 2020_Tu | H |
| 2019_Wu | U |
| 2009_Zhao | U |
| 2006_Coleman | H |
| 1999_Naylor | H |
| 2013_Altfeld | H |
| 2015_Balaban | H |
| 1996_Bostrom | H |
| 2014_Kangovi | H |
| 2009_Courtney | L |
| 2012_Li | H |
| 2012_Davis | L |
| 2002_Harrison | L |
| 2004_Boter | L |
| 2013_Lainscak | H |
| 2003_Naunton | U |
| 2014_Burns | H |
| 2021_Anabanula | H |
| 2020_Carroll | U |
| 2019_Chaundry | H |
| 2020_Jan | U |
| 2020_Melamed | H |
| 2020_Myc | H |
| 2018_Rosovsky | U |
| 2021_Wright | H |
| 2019_Xenos | U |
| 2007_Aldamiz | U |
| 2012_Angermann | L |
| 2014_deSouza | H |
| 2011_Domingues | H |
| 2005_Ducharme | U |
| 2005_Dunagan | U |
| 2008_Kwok | L |
| 2003_Laramee | U |
| 2006_Linne | U |
| 2004_Naylor | L |
| 2006_Nucifora | H |
| 2016_Ong | L |
| 2001_Pugh | H |
| 2002_Riegel | U |
| 2006_Riegel | U |
| 2016_Ritchie | L |
| 2008_Schwarz | H |
| 2005_Thompson | U |
| 2019_Van Spall | U |
| 2002_Kasper | U |
| 2002_McDonald | H |
| 2019_Negarandeh | U |
| 2004_Sethares | U |
| 2001_Barth | H |
| 2003_Stomberg | U |
| 2012_Barker | U |
| 2008_Falces | U |
| 2006_Lopez | H |
| 2004_Tsuyuki | U |
| 2009_Dar | H |
| 2019_Huynh | U |
| 2012_Liu | U |
| 2018_Chen | U |
| 2007_Albert | U |
| 2013_Sales | H |
| 2016_Al-Rajhi | SERIOUS |
| 2012_Chaboyer | SERIOUS |
| 2006_Chaboyer | SERIOUS |
| 2008_Eliott | SERIOUS |
| 2018_So | SERIOUS |
| 2010_Harrison | SERIOUS |
| 2008_Pirret | CRITICAL |
| 2017_Bergamasco | SERIOUS |
| 2016_Stelfox | SERIOUS |
| 2016_Choi | CRITICAL |
| 2015_Martin | CRITICAL |
| 2013_Al-Qahtani | CRITICAL |
| 2010_Williams | CRITICAL |
| 2008_Baxter | CRITICAL |
| 2004_Garcea | CRITICAL |
| 2003_Ball | CRITICAL |
| 2015_Bell | L |
| 2001_Coleman | SOME CONCERNS |
| 2002_Dally | SOME CONCERNS |
| 2020_Finkelstein | L |
| 2020_Katzelnick | SOME CONCERNS |
| 2020_Kelley | L |
| 2017_Lin | SOME CONCERNS |
| 2010_McCall_a | SOME CONCERNS |
| 2010_McCall_b | SOME CONCERNS |
| 2010_McCall_c | SOME CONCERNS |
| 2010_McCall_d | LOW |
| 2011_McCall | SOME CONCERNS |
| 2020_Powers | SOME CONCERNS |
| 2020_Raven | SOME CONCERNS |
| 2017_Seaberg | SOME CONCERNS |
| 2008_Shumway | H |
| 2006_Sledge | L |
| 2013_Urato | SOME CONCERNS |
| 2018_Yoon | SOME CONCERNS |
| 2009_Thomas | H |
| 2010_Morrison | U |
| 2011_Lilly | L |
| 2000_Rosenfeld | L |
| 2004_Breslow | U |
| 2010_McCambridge | U |
| 2013_Sadaka | H |
| 2014_Fortis | H |
| 2017_Panlaqui | L |
| 2012_Kohl | H |
| 2014_Nassar | L |
| 2012_Willmitch | H |
| 2016_Kahn | U |
| 2018_Biese | L |
| 2015_Cossette | SOME CONCERNS |
| 2014_Englander | L |
| 2018_Finlayson | L |
| 2014_Goldman | SOME CONCERNS |
| 1996_Griffiths | H |
| 2001_Griffiths | SOME CONCERNS |
| 2009_Jack | L |
| 2001_Steiner | SOME CONCERNS |
| 2019_Weiss | SOME CONCERNS |
| 2012_Wong | H |
| 2014_Wong | SOME CONCERNS |
| 2002_Hermiz | H |
| 2002_Stewart | H |
| 2003_Young | H |
| 2006_Casas | H |
| 2006_Latour | H |
| 2007_Kwok | H |
| 2010_Rytter | H |
| 2011_Leventhal | H |
| 2012_Stewart | H |
| 2013_Makaya | H |
| 2014_Dhalla | H |
| 2015_Lee | H |
| 2015_Stewart | H |
| 2016_Buurman | H |
| 2016_Wong | H |
| 2017_Zimmermann | H |
| 2018_McWilliams | H |
| 2010_Adam | H |
| 2017_Capp | SOME CONCERNS |
| 2012_Crane | H |
| 2012_DeHaven | H |
| 2018_Durfee | SOME CONCERNS |
| 2013_Enard | H |
| 2020_Harrison | SOME CONCERNS |
| 2016_Horn | H |
| 2019_Kimmey | SOME CONCERNS |
| 2013_McCormack | H |
| 2014_Navrati | SOME CONCERNS |
| 2018_Peikes | SOME CONCERNS |
| 2019_Peikes | SOME CONCERNS |
| 2019_Schnickendanz | SOME CONCERNS |
| 2018_Sevak | SOME CONCERNS |
| 2011_Shah | SOME CONCERNS |
| 2018_Thompson | H |
| 2019_Valluru | H |
| 2018_Vickery | SOME CONCERNS |
| 2015_Weerahandi | SOME CONCERNS |
| 2015_Adamuz | L |
| Arendts | L |
| 2018_Auger | L |
| 2008_Balaban | H |
| 2017_Barfar | L |
| 2020_Barker | H |
| 2021_Bewazeer | L |
| 2019_Bloodworth | L |
| 2019_Bloom | H |
| Blum | H |
| 2018_Bonetti | H |
| Bonnet-Zamponi | H |
| 2016_Bonsack | L |
| 2021_Bouchard | L |
| 2019_Bruhwiler | H |
| 2019_Chen | L |
| 2021_Coskun | L |
| 2018_Dalal | H |
| 2020_Danielsen | L |
| Dawes | L |
| 2021_Devore | L |
| 2019_Edey | H |
| 1993_Evans | H |
| 2014_Farris | L |
| 2020_Finn | L |
| 2020_Gardner | H |
| 2015_Gharadi | L |
| 2021_Gilbert | L |
| 2022_Gillard | L |
| 2009_Gillespie | L |
| 2018_Graabaek | L |
| 2014_Gurwitz | L |
| 2021_Habib | L |
| Hanrahan | L |
| 2019_Heaton | L |
| 2019_Hegelund | H |
| 2020_Hegelund | H |
| 2016_Hengartner | L |
| 2020_Hosein | L |
| 2022_Idraratna | L |
| 2022_Jayaram | L |
| 2014_Jennings | L |
| 2020_Karaoui | L |
| 1987_Kennedy | H |
| 2017_Ko | L |
| 2022_Kowalkowski | H |
| 2012_Kripalani | L |
| 2013_Lainscack | L |
| Latour | H |
| 2016_Lavesen | H |
| 2020_Lea | L |
| 2011_Legrain | L |
| 2018_Levine | L |
| 2014_Li | L |
| 2018_Li | L |
| 2020_Liu | L |
| 2019_Lockwood | L |
| 2019_McWilliams | L |
| 2020_Mehta | L |
| Meisinger | L |
| 1992_Moher | H |
| 2001_Nazareth | H |
| 2020_Noel | L |
| 2018_O'Connell | L |
| Odeh | L |
| 2019_Oscalices | L |
| 2020_Ougrin | L |
| 2002_Paradesus | H |
| 2018_Parsons | H |
| 2020_Parsons | L |
| 2020_Pourrat | L |
| 2019_Qian | H |
| 2018_Ravn-Nielson | L |
| 2004_Reynolds | H |
| 1993_Rich | H |
| 1995_Rich | H |
| 2017_Sahota | L |
| 2018_Salameh | L |
| 2018_Salmany | H |
| 2017_Sanatana | L |
| 2017_Shahronki | H |
| 2000_Shaw | H |
| 2019_Strano | L |
| 2018_Sudas Na | L |
| 2015_Thygesen | L |
| 2012_Tomita | H |
| Utens | L |
| 2019_van der Heijden | H |
| 2017_Vesterby | L |
| Webster | L |
| 2015_Wong | L |
| 2018_Xie | L |
| 2019_Xu | L |
| 2020_Yiadom | L |
| 2020_Yin | L |
| 2017_Zhang | H |
| Boult | H |
| Ekdahl | H |
| Gagnon | H |
| Imhof | H |
| Kirchberger | H |
| Linden | H |
| Newcomer | H |
| Parry | H |
| Shannon | H |
| Note. H: High risk; L: Low risk; U: Unclear risk | |

**Appendix Figures 4-5 & 6-7.** Meta-analytic estimates regarding the effectiveness of intermediate care interventions in reducing ED/hospital re-admissions*. Meta-analytic estimates regarding the effectiveness of intermediate care interventions in reducing the risk of mortality and increasing the QoL**,***.

| \| ED/hospital re-admissions \| \| --- \| \| 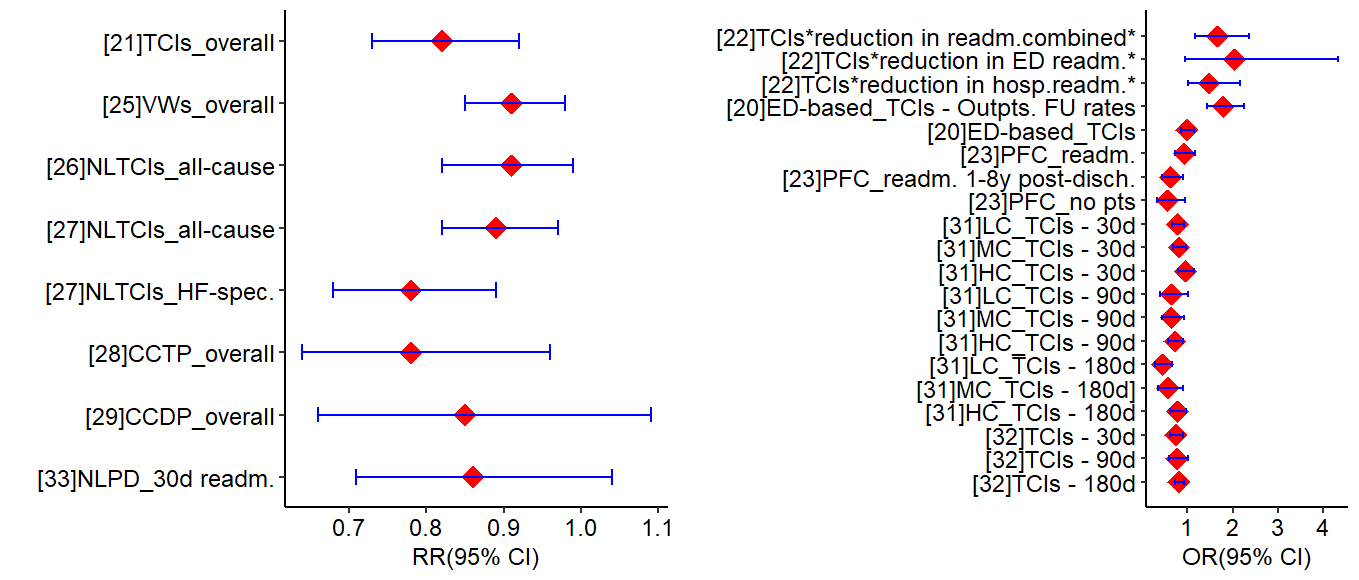 \| | |
| --- | --- | --- | --- |
| Mortality | QoL |
| 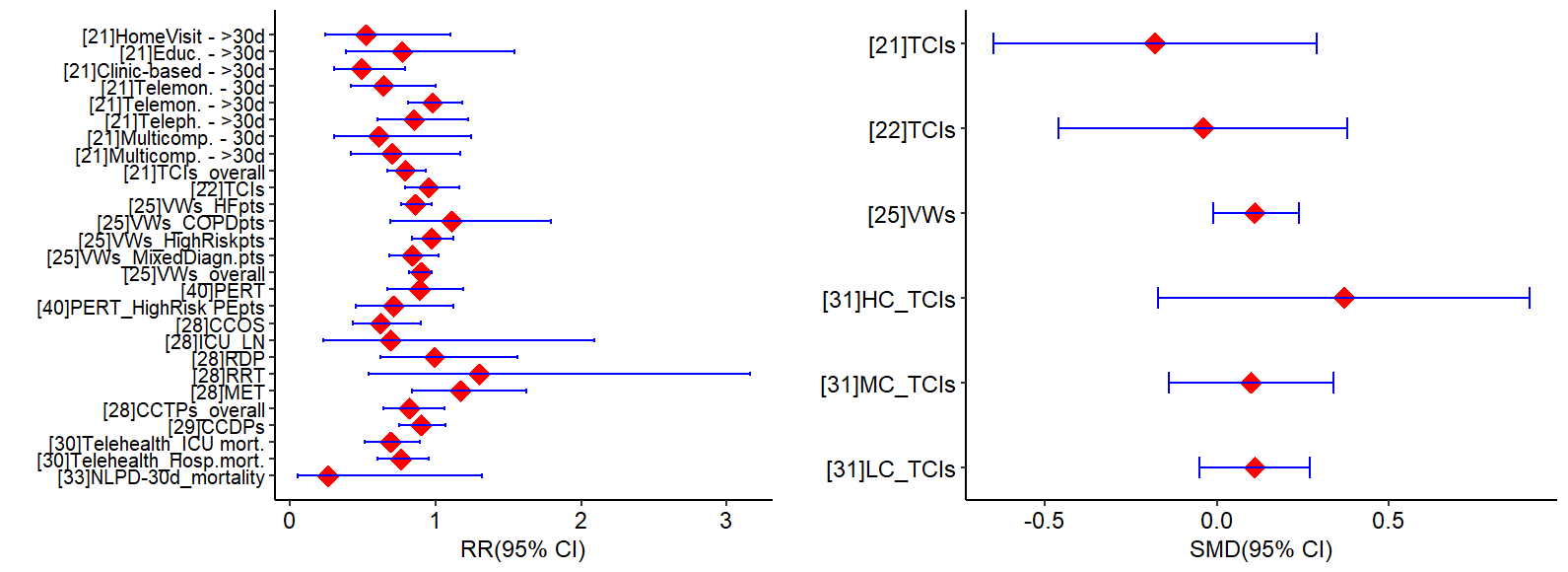 | |
| ***Note***. CCDPs: critical care stepdown programmes; CCTPs: Critical care transition programmes; CI: confidence interval; COPD: chronic obstructive pulmonary disease; HF: heart failure; ICU: intensive care unit; MET: Medical emergency team; NL: nurse-led; NLPD: nurse-led peri-discharge; PERTs: pulmonary embolism response teams; RDP: redesigned discharge process; RR: risk ratio; RRT: Rapid response team; TCIs: transitional care interventions; VW: virtual ward.  *Values <1 indicate lower probability to be readmitted to ED/hospital, favouring the intermediate care interventions  **Values <1 indicate lower probability of mortality, favouring the intermediate care interventions.  ***Values >0 indicate better QoL, favouring the intermediate care interventions. | |
